# Dissecting Quantitative Trait Loci for Boron Efficiency across Multiple Environments in *Brassica napus*

**DOI:** 10.1371/journal.pone.0045215

**Published:** 2012-09-24

**Authors:** Zunkang Zhao, Likun Wu, Fuzhao Nian, Guangda Ding, Taoxiong Shi, Didi Zhang, Lei Shi, Fangsen Xu, Jinling Meng

**Affiliations:** 1 National Key Laboratory of Crop Genetic Improvement, Huazhong Agricultural University, Wuhan, Hubei, China; 2 Microelement Research Centre, Huazhong Agricultural University, Wuhan, Hubei, China; United States Department of Agriculture, Agricultural Research Service, United States of America

## Abstract

High yield is the most important goal in crop breeding, and boron (B) is an essential micronutrient for plants. However, B deficiency, leading to yield decreases, is an agricultural problem worldwide. *Brassica napus* is one of the most sensitive crops to B deficiency, and considerable genotypic variation exists among different cultivars in response to B deficiency. To dissect the genetic basis of tolerance to B deficiency in *B. napus*, we carried out QTL analysis for seed yield and yield-related traits under low and normal B conditions using the double haploid population (TNDH) by two-year and the BQDH population by three-year field trials. In total, 80 putative QTLs and 42 epistatic interactions for seed yield, plant height, branch number, pod number, seed number, seed weight and B efficiency coefficient (BEC) were identified under low and normal B conditions, singly explaining 4.15–23.16% and 0.53–14.38% of the phenotypic variation. An additive effect of putative QTLs was a more important controlling factor than the additive-additive effect of epistatic interactions. Four QTL-by-environment interactions and 7 interactions between epistatic interactions and the environment contributed to 1.27–4.95% and 1.17–3.68% of the phenotypic variation, respectively. The chromosome region on A2 of *SYLB-A2* for seed yield under low B condition and *BEC-A2* for BEC in the two populations was equivalent to the region of a reported major QTL, *BE1*. The *B. napus* homologous genes of Bra020592 and Bra020595 mapped to the A2 region and were speculated to be candidate genes for B efficiency. These findings reveal the complex genetic basis of B efficiency in *B. napus*. They provide a basis for the fine mapping and cloning of the B efficiency genes and for breeding B-efficient cultivars by marker-assisted selection (MAS).

## Introduction

Boron (B) is an essential micronutrient for the growth and development of higher plants [1]. A key role of B in plants is to cross-link rhamnogalacturonan II (RG-II) in the cell wall to form a dimer (RG-II-B-RG-II), which is important for both the formation and the structural integrity of the cell wall [2], [3]. It has also been reported that B seems to play important roles in many diverse processes in vascular plants, such as root elongation, sugar translocation, carbohydrate metabolism, nucleicacid synthesis, pollen tube growth and nitrogen fixation [4], [5].

It is well known that plants absorb B from soil in the form of boric acid. It has been widely thought that passive diffusion is a major mechanism of B trans-membrane transport, with B translocation in plants being a passive process through the transpiration stream [6]. However, genotype differences in response to B deficiency, or in uptake and utilization of B among species, suggest that energy-dependent active transport and channel-mediated diffusion are involved in B transport [7]. Several transporters and channel proteins for B have been discovered in plants. The *AtBOR1* gene from *Arabidopsis* shows the characteristics of an efflux-type transporter for xylem loading of B [8] and is mainly regulated by post-transcriptional mechanisms [9]. Four *BOR1* homolog have been validated in rice, and *OsBOR1* is required for efficient uptake of B and xylem loading under low B conditions [10]. Sun et al. (2011) cloned six *BOR1*-like homologs in *B. napus*: *BnBOR1;3a* and *BnBOR1;3c* showed ubiquitous expression in all of the investigated tissues, whereas the other four genes showed similar tissue-specific expression profiles. However, the expression of *BnBOR1;1c* and *BnBOR1;2a* was clearly induced by B deficiency [11]. Another type of B transporter, *ATR1* in yeast, is up-regulated at the transcriptional level by B [12]. Some members of the major intrinsic protein (MIP) family have been identified as boric acid channels in plants. *NIP5;1* and *NIP6;1* function as channel proteins for boric acid transport in *Arabidopsis* [13], [14]. Kasajima et al (2010) first identified the transcription factor gene *WRKY6* as essential for root growth under B deficiency [15]. In general, plants growing in nutrient-deficient conditions up-regulate the expression of transporters and thereby increase transport of the deficient nutrient [4]. The induction of transporter expression in plants suggests that further enhancement of transporter genes would result in addition transport capacity and allow plants to perform better under conditions of limited nutrients. Several examples of enhanced B transport activity and better growth (more biomass or seed yield) due to the increased expression of transport genes in *Arabidopsis* have been reported. Over-expression of *AtBOR1* increased the seed yield of *Arabidopsis* under low B condition [16]. Enhanced expression of *AtNIP5;1* can significantly promote B uptake under low B stress and increase seed yield [17]. However, there are few reports on B uptake and transport-related genes and on the relationship between these genes and seed yield in *B. napus*.

*B. napus* (genome AACC, 2n = 38), plants are commonly used to derive food oil for humans and as a new type of bio-fuel [18]. After soybeans, *B. napus* is the second most important oilseed crop in the world. However, *B. napus* requires higher levels of B than do other species to maintain normal growth and shows high sensitivity to B deficiency [4]. B deficiency causes severe reduction in yield and can even cause a lack of seed setting, which is becoming an important limiting factor for *B. napus* growth in a large area of the world [19]. B deficiency can be alleviated by the application of B fertilizers. However the extreme use of B fertilizers could cause environmental problems. Moreover, B ore resources are limited. The various *B. napus* cultivars have genetic variations that affect their B efficiency. Thus, the development of cultivars with enhanced B-use efficiency would be a more efficient way of genetic improvement. Unveiling the genetic mechanisms involved in plant responses to B deficiency will be the first important step to solve this issue.

Most nutritional traits of crop plants are quantitative and have a complex genetic basis [20], [21]. Quantitative trait locus (QTL) mapping has proved to be a powerful genetic approach for dissecting the genetic mechanism of complex traits [22], [23]. In some plants, a number of QTLs for complex traits have been mapped [24], [25]. QTLs associated with yield and yield-related traits in *B. napus* have been mapped, including plant height [26], yield and yield components [27], [28], seed weight [29], and other complex traits [30]. Xu et al. (2001) first revealed that the B efficiency trait is a quantitative trait. A major QTL (*BE1*) and three minor QTLs for B efficiency were detected from an F_2_ population derived from a cross between two cultivars in *B. napus* [21]. Furthermore, *BE1* was located in a narrow interval using an F_2;3_ family population derived from a cross between the two cultivars described above [31]. A new locus, *BnBE2*, that controls B use efficiency was validated by bulked segregant analysis (BSA) in a back crossed population of *B. napus* [32]. However, it is still difficult to accurately predict potential candidate genes involved in B efficiency. Additionally, only a few B efficiency loci (rarely >10) have been found to be associated with seed yield or biomass; thus, the genetic architecture of B efficiency remains ambiguous.

B efficiency can be defined as the ability of a genotype to grow well and produce a high yield under B-deficient conditions. The objective of this study is to investigate natural variations for yield and yield-related traits under different B conditions and dissect the genetic basis of B efficiency in *B. napus* using two DH populations, TNDH and BQDH, in five environments. The TNDH population was derived from the F_1_ progeny of a cross between Ningyou7 (B-efficient) and Tapidor (B-inefficient). Its genetic linkage map has become an international reference map for *B. napus* (http://brassica.bbsrc.ac.uk) [33], [34], [35]. The BQDH population was derived from the F1 progeny of a cross between QY10 (B-efficient) and Bakow (B-inefficient) [21]. In this study, QTLs associated with seven yield and yield-related traits were identified using the two DH populations under low and normal B field treatments in three growth seasons. Genes involved in B uptake and utility, yield and yield-related traits in *Arabidopsis* were mapped to the QTL interval. Some QTLs expressed during a low B supply may provide valuable information for improving the seed yield of *B. napus* in soils with low B levels via MAS.

## Results

### Construction of the genetic linkage map

A genetic map, named the BQDH map, was constructed based on the BQDH population and spanned 19 linkage groups corresponding to 19 chromosomes in *B. napus* named as A1-A10 and C1-C9 according to the new standardized nomenclature for *B. napus* linkage groups (http://www.brassica.info/resource/maps/lg-assignments.php). The BQDH genetic map comprises a total of 486 molecular markers, including 468 SSR, 9 GBM and 7 SRAP markers. This map covers a total length of 1873.9 cM with an average interval of 3.86 cM between adjacent markers. The largest distance between two adjacent markers is 41.98 cM on chromosome C3. The length of the 19 linkage groups varies from 23.31 to 194.71 cM (Table S1).

A comparative analysis between the BQDH and TNDH genetic map by common markers indicated that the BQDH genetic map was collinear with the TNDH genetic map. The two genetic maps were employed in the QTL analysis.

### Phenotypic variation and genetic correlation analysis among traits

**Figure 1:**
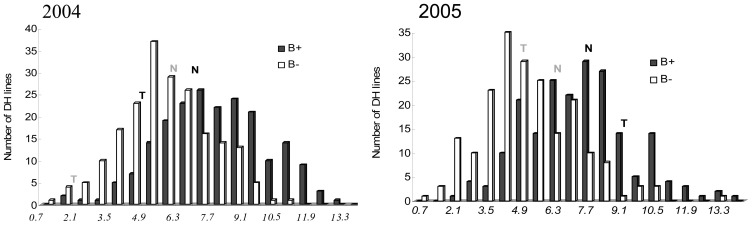
Frequency distribution of seed yield for the TNDH population and its parents at low B (B−) and normal B (B+) conditions in 2004 and 2005 (T: Tapidor; N: Ningyou7; low B grey; normal B black).

In the TNDH population, seed yield (SY) under low B (LB) and normal B (NB) conditions, as well as the B efficiency coefficient (BEC), were investigated in a two-year field trial (Table 1). Considerable variation was observed for SY and the BEC between the two parents and among the DH lines during the two years at both B conditions. The B-efficient parent Ningyou7 (NY7) showed a higher SY and larger BEC than the B-inefficient parent Tapidor under the LB condition. In the two trials, SY displayed a continuous normal distribution in the TNDH population, and significant transgressive segregation was observed under both B conditions (Fig. 1).

**Table 1:** Mean values and ranges of seed yield and BEC traits in the parental and DH lines of TN population.

Traits	Year	Parents				DH lines					
		LB		NB		LB			NB		
		NY7	Tapitor	NY7	Tapitor	Mean	Range	CV(%)	Mean	Range	CV(%)
**SY(g/plant)**	2004	11.060	4.840	14.750	9.420	4.260	(0.760–9.931)	48.0	7.252	(0.745–13.356)	33.6
	2005	9.100	3.820	12.770	9.990	5.640	(0.912–10.443)	35.5	6.994	(0.881–12.882)	28.9
**BEC**	2004	0.750	0.514			0.607	(0.078–1.339)	49.5			
	2005	0.713	0.382			0.804	(0.210–1.299)	29.7			

Note: SY, seed yield; BEC, B efficiency coefficient; CV, coefficient of variation.

**Figure 2:**
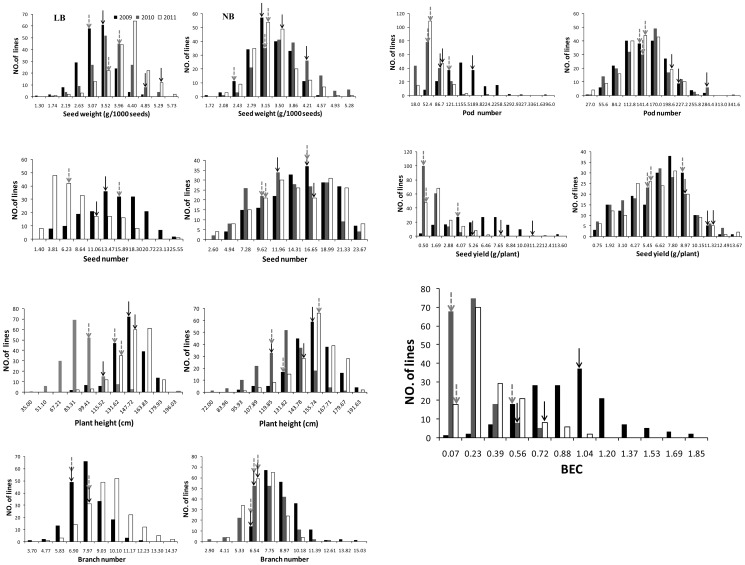
Frequency distributions of seed yield and yield related traits in the BQDH population under low B (left) and normal B (right) levels and BEC in the three-year field trials. Solid arrows indicate QY10, and dashed arrows indicate Bakow.

In the BQDH population, a broad variation for seed yield and six yield-related traits was observed among the parents and DH lines under LB and NB conditions (Table 2). In general, the B-efficient parent Qingyou 10 (QY10) was significantly taller plant (PH 114.9–142.4 cm) and had a heavier seed weight (SW 3.55–5.24 g/1000), more seeds (SN 10.1–14.0), more pods (83.5–191.9), a better yield (5.135–10.962 g) and a larger BEC (0.627–0.950) than the B-inefficient parent Bakow under the LB condition. Compared to the NB condition, both parents and the DH lines showed smaller phenotypic values under the LB condition for all of the six measured traits, except for BN and SW. The BEC value was higher in QY10 than in Bakow in the three trials. All traits were highly variable, SY and the BEC were the most variable traits, with CVs of 37.1%–114.1%, and these traits showed more variability under the LB condition than under the NB condition. In the three field trials, all seven traits showed continuous phenotypic variation and significant transgressive segregation in both directions, implying that multiple genes were involved (Fig. 2). The ANOVA results suggest that genotype, B level, year and the interactions among these variable had significant effect on the six measured traits (*p*<0.001).

**Table 2:** Mean values and ranges of seed yield and yield-related traits in the parental and DH lines of BQDH population.

Traits	Year	Parents				DH lines					
		LB		NB		LB			NB		
		QY10	Bakow	QY10	Bakow	Mean	Range	CV(%)	Mean	Range	CV(%)
**PH(cm)**	2009	142.4bcd	134.1de	151.9ab	134.4de	138.2	(82.0–179.4)	13.1	147.2	(90.3–186.5)	11.6
	2010	114.9f	98.3g	125.3ef	114.9f	81.7	(30.0–141.3)	23.4	123.5	(66.8–169.1)	14.8
	2011	140.0cd	135.0de	146.7abc	154.9a	141.1	(69.9–191.0)	13.1	149.7	(87.6–185.1)	11.5
**BN**	2009	7.2bc	7.1bcd	6.9bcd	6.8bcd	7.4	(3.3–11.2)	17.6	8.2	(5.8–14.5)	16.8
	2010			6.8bcd	6.5bcd				6.9	(2.4–11.8)	22.4
	2011	7.6ab	8.4a	6.0d	6.1cd	9.0	(3.9–14.0)	18.7	6.5	(3.4–10.2)	19.3
**SN(/pod)**	2009	14.0b	15.3b	17.3a	17.5a	13.3	(2.7–24.4)	37.0	14.3	(3.1–25.0)	33.1
	2010			11.1c	9.9c				12.2	(2.0–22.7)	39.6
	2011	10.1c	5.8d	17.4a	9.9c	7.04	(0.20–23.20)	66.2	13.52	(1.59–24.36)	40.1
**PN(/plant)**	2009	191.9c	130.3ef	225.5b	139.3cd	142.8	(22.4–380.0)	41.0	133.8	(22.4–264.9)	35.1
	2010	83.5g	40.3h	270.5a	129.1ef	45.5	(2.0–198.7)	75.4	135.0	(14.4–329.0)	41.4
	2011	102.8fg	51.3h	185.0c	148.2de	47.3	(3.4–154.3)	57.8	128.4	(21.3–236.8)	34.7
**SW(g/1000)**	2009	3.55de	3.02f	3.26ef	2.54g	3.035	(1.200–4.570)	17.6	3.111	(1.600–4.483)	15.4
	2010	4.84b	3.79de	4.20c	3.30ef	3.503	(1.613–5.053)	17.8	3.485	(1.917–5.163)	18.0
	2011	5.24a	3.72d	3.58de	3.06f	3.933	(1.641–5.633)	16.6	3.153	(1.830–5.100)	17.2
**SY(g/plant)**	2009	10.962a	4.582d	11.541a	9.426b	5.219	(0.230–13.107)	52.1	6.044	(0.505–13.175)	43.2
	2010	5.135d	0.321e	8.191c	5.311d	0.745	(0.006–4.717)	114.1	5.680	(0.407–12.210)	48.8
	2011	7.403c	0.712e	11.289a	5.068d	1.474	(0.013–7.618)	97.9	5.736	(0.256–12.598)	46.3
**BEC**	2009	0.950	0.486			0.854	(0.029–1.784)	37.1			
	2010	0.627	0.124			0.140	(0.001–0.666)	98.5			
	2011	0.656	0.140			0.265	(0.003–0.990)	79.8			

Note:

SY, seed yield; BEC, B efficiency coefficient; PH, plant height; BN, branch number per plant; SN, seed number; PN, pod number per plant; SW, seed weight; LB, low B condition; NB, normal B condition; CV, coefficient of variation. Different small letters indicate significant difference at the level of *P*<0.05.

For all environments, significantly positive correlation was observed between the SY under the LB condition and the BEC in the two populations (Table S2). These results indicate that SY and the BEC may be controlled by some common genetic determinants for tolerance in a B-limited environment. In the BQDH population, significant positive correlations were observed between SY and all traits except BN and between the BEC and all traits except PH and BN under the LB condition. Under the NB condition, significant negative correlations were observed between the BEC and all traits, except SW and SN, and between SY and SW. Significant positive correlations were observed between SY and PH and between SN and PN.

### Detection of putative QTLs in the TNDH and BQDH populations

Seed yield under LB and NB conditions and BEC were used to detect QTLs in the TNDH population. A total of 10 putative QTLs (6 for SY, 4 for BEC) during the two-year trials were detected in the 6 linkage groups, A2, A4, A6, A9, C3 and C4 (Table 3). The phenotypic variation explained (PVE) for the QTLs ranged from 5.7% to 18.5%. One of the four putative QTLs (*SYLB-A6*) for SY under the LB condition was detected across two years and explained 6.1–6.4% of the phenotypic variation of the favorable alleles from Tapidor. One of the four putative QTLs for the BEC (*BEC-A2*) was detected across two years for the favorable alleles from NY7. Additionally, two putative QTLs (*SYLB-A2a* and *SYLB-A2b*) for the SY under the LB condition and 2 putative QTLs (*SYNB-A2* and *SYNB-A9*) for the SY under the NB condition showed positive effects, indicating that the favorable allele was from NY7.

**Table 3:** Putative QTLs for seed yield and BEC traits in the TNDH population under different B conditions.

Trait^a^	Chr.	QTL^b^	Add.^c^	2004			2005		
				PVE(%)^d^	CI^e^	Peak^f^	PVE(%)	CI	Peak
BEC	A2	*BEC-A2*	+	8.3	64–72	65.5	7.7	65–100	66.4
	A4	*BEC-A4*	−	8.4	103–120	110.5			
	A6	*BEC-A6*	−				10.2	15–26	18
	C3	*BEC-C3*	−	10.3	114–123	120			
**QTLs detected under low boron (LB) condition**									
SY	A2	*SYLB-A2a*	+				8.1	35–46	42
		*SYLB-A2b*	+	18.5	60–81	70			
	A6	*SYLB-A6*	−	6.4	13–30	23	6.1	13–29	23
	C4	*SYLB-C4*	−	5.7	75–80	77			
									
**QTLs detected under normal boron (NB) condition**									
SY	A2	*SYNB-A2*	+	7.3	33.8–38.1	36	7.4	32.7–45.7	40
	A9	*SYNB-A9*	+	8.1	80.1–85.2	83			

Note:

a , short names of the traits: BEC, boron efficiency coefficient; SY, seed yield; SW, seed weight; PH, plant height; SN, seed number; PN, pot number; BN, branch number.

b , Nomenclature for QTL: an trait abbreviation following a boron-level designator (LB, low B level; NB, normal B level), a hyphen (-), chromosome (A1-A10 or C1-C9) on which the QTL located and the serial letter (a, b, c…) in the same linkage group.

c , Additive effect. Positive additive effects are associated with increased effects from B-efficient parent allele, and negative additive effects are associated with increased effects from B-inefficient parent allele.

d , Percentage of phenotypic variation explained (PVE) by each identified QTL.

e , The 2-LOD confidence interval (CI) of QTL, given in cM.

f , The peak position is denoted by the number in parentheses.

A total of seventy putative QTLs under both B conditions were identified contributing 4.15%–23.16% of the phenotypic variation in the BQDH population. These QTLs were distributed among 15 linkage groups, which included 13 for SW, 15 for PH, 9 for SN, 10 for PN, 9 for BN, 9 for SY and 5 for the BEC (Table 4). Interestingly, the A and C genome shared an equal number of QTLs with 34 and 36 QTLs, respectively.

**Table 4:** Putative QTLs for seed yield and yield-related traits in the BQDH population under different B conditions.

Trait^a^	Chrom.	QTL^b^	Add.^c^	2009			2010			2011		
				PVE(%)^d^	Cie	Peak^f^	PVE(%)	CI	Peak	PVE(%)	CI	Peak
BEC	A2	*BEC-A2*	+				6.80	71.2–76.4	72.81			
	A7	*BEC-A7a*	+							8.25	0–14.8	5.01
		*BEC-A7b*	+				11.24	19.4–38	27.41			
	C3	*BEC-C3*	−	11.44	68.9–75.3	71.91						
	C8	*BEC-C8*	+							7.33	11.9–23.4	16.41
**QTLs detected under low boron (LB) condition**												
SW	A1	*SWLB-A1a*	−				6.00	35.8–46.5	38.31			
		*SWLB-A1b*	+	6.49	68.8–78.1	73.11						
	A4	*SWLB-A4*	+	8.40	7.7–22.3	11.61						
	A7	*SWLB-A7a*	+				6.20	19.4–41	29.71	6.02	34.9–41.7	37.21
		*SWLB-A7b*	−	7.18	75.1–77.9	76.21	6.03	71.9–87.9	75.11	9.07	71.9–77.9	76.21
	C6	*SWLB-C6*	+	6.96	29.5–39.7	31.51						
	C8	*SWLB-C8*	+				7.05	11.9–20.2	13.61			
PH	A3	*PHLB-A3a*	−	11.89	19.8–23	21.41				6.09	22.5–25	23.01
		*PHLB-A3b*	−	10.51	31.3–34.1	32.31				11.93	29.9–35.9	34.31
	A7	*PHLB-A7*	+	5.25	58.4–73.7	68.71						
	C3	*PHLB-C3a*	−				8.80	0–23.4	12.01			
		*PHLB-C3b*	−							5.52	26.3–39.9	31.21
	C4	*PHLB-C4*	+	6.78	37.8–48.6	43.81						
SN	A4	*SNLB-A4*	−							8.26	7.9–15.9	11.61
	C6	*SNLB-C6*	−	20.05	5.3–17.8	9.31						
	C9	*SNLB-C9*	+							11.20	78.5–85.2	84.21
PN	A2	*PNLB-A2*	−				10.32	78–84.4	83.01			
	A6	*PNLB-A6*	+	8.32	6.5–33.8	26.81						
	A7	*PNLB-A7a*	+				8.21	6.9–29.5	21.41			
		*PNLB-A7b*	+							5.64	29.6–56.2	44.71
		*PNLB-A7c*	+				5.77	85.4–99.2	98.21			
	C4	*PNLB-C4*	−							6.01	76.3–83.2	81.31
	C5	*PNLB-C5*	−	6.55	41.2–64.5	54.31						
BN	A1	*BNLB-A1*	+	6.35	39–56.3	48.51						
	C9	*BNLB-C9*	−	6.01	39.6–53	41.81				7.09	39.6–48.9	41.81
SY	A2	*SYLB-A2*	+				9.11	71.6–83	76.4			
	A7	*SYLB-A7*	+				11.24	19.4–38	27.41	6.73	15.6–29.6	20.41
	C3	*SYLB-C3*	−	7.19	64.9–77.2	68.91						
	C4	*SYLB-C4*	−							7.85	76.3–104.2	84.21
	C7	*SYLB-C7*	+							8.09	22.7–53.9	44.11
												
**QTLs detected under normal boron (NB) condition**												
SW	A7	*SWNB-A7*	−	14.41	73.8–77.9	76.21	12.23	77.9–94.3	79.91	9.10	76.2–80.8	77.91
	A9	*SWNB-A9*	−	5.20	65–80.7	69.01						
	A10	*SWNB-A10*	+				9.11	19–41.5	32.21	7.17	18.2–41.4	29.91
	C6	*SWNB-C6a*	+				16.92	4.5–21.4	13.31			
		*SWNB-C6b*	+	12.24	26.8–35.4	30.51				10.97	29.5–37	30.61
	C9	*SWNB-C9*	+	5.43	11.1–37.8	32.31	5.72	18.2–36.9	31.41	8.43	22.7–32.3	30.41
PH	A3	*PHNB-A3a*	−	12.30	18.1–24.2	21.41	9.66	18.8–24.3	23.01	5.16	22.4–24.5	23.01
		*PHNB-A3b*	−	7.42	31.3–32.8	32.31	5.10	31.3–33.8	32.31	9.83	29.2–36.1	34.31
	A7	*PHNB-A7*	+	7.44	77.9–91.8	80.81						
	C3	*PHNB-C3*	−	5.12	25.8–45.5	35.21				5.45	26.3–45.8	36.21
	C4	*PHNB-C4*	+	8.61	60–61.4	60.91						
	C5	*PHNB-C5*	−	7.10	8.1–38.6	17.41	4.75	8.2–38.6	24.61			
	C6	*PHNB-C6*	−							5.35	0–4.1	0.01
	C9	*PHNB-C9a*	−	4.15	8.1–37.8	33.91						
		*PHNB-C9b*	−				6.37	58.2–77.1	65.61			
SN	A2	*SNNB-A2*	−				6.25	74–85.1	83.01			
	A6	*SNNB-A6*	+				8.64	76–97.5	88.01			
	A7	*SNNB-A7*	+	7.95	80.4–98.2	90.81						
	C6	*SNNB-C6a*	−	23.26	5.3–17.2	9.31	16.55	8–24.5	17.31	15.14	6.9–22.2	15.31
		*SNNB-C6b*	−				13.22	30.6–37.4	31.51	8.93	30.6–37.6	32.51
	C9	*SNNB-C9*	+				6.21	70.3–78.2	73.01	5.06	66.9–78.2	73.21
PN	A6	*PNNB-A6*	+	8.29	74.5–99	91.01						
	C6	*PNNB-C6*	−	5.68	17.2–26.8	24.51	10.19	4.1–21.4	8.31			
	C9	*PNNB-C9*	−	12.67	30.5–33.9	32.31	8.87	13.6–39.6	31.41	6.70	9.5–40.4	33.31
BN	A6	*BNNB-A6*	+	6.21	61.4–74	61.71						
	A7	*BNNB-A7*	+				5.01	61.3–75.1	71.91			
	C3	*BNNB-C3a*	+	7.46	15.5–26.3	21.91						
		*BNNB-C3b*	+				5.25	76.5–92.8	80.41			
	C6	*BNNB-C6*	−				9.26	12.6–29.3	25.51			
	C9	*BNNB-C9a*	−	9.61	30.3–33.9	31.41	16.98	32.3–36.9	33.31			
		*BNNB-C9b*	−	10.71	37.8–47.5	41.41				10.97	39.6–43.5	41.81
SY	A1	*SYNB-A1*	−				12.07	37.4–44.5	40.31	7.18	28.7–49.6	39.31
	A7	*SYNB-A7*	+	7.95	19.4–41.4	26.41						
	C6	*SYNB-C6a*	−				13.92	5.3–24.5	15.31	8.63	4.1–21.6	8.31
		*SYNB-C6b*	−	10.18	22.2–30.6	25.51						

a–f See footnotes of Table 3 for explanations.

Thirty putative QTLs were detected in thirteen linkage groups under the LB condition (Table 4). One QTL (*SWLB-A7b*) was detected across three years, contributing 6.03–9.07% of the phenotypic variation. Five QTLs (*SWLB-A7a*, *PHLB-A3a*, *PHLB-A3b*, *BNLB-C9* and *SYLB-A7b*) were detected across two years, contributing 6.01–11.93% of the phenotypic variation. Seven putative QTLs for seed yield in A2, A7, C3, C4 and C7 were identified. Among these QTLs, the QTL *SYLB-A7*, which was detected across two years with favorable alleles from QY10, co-localized with the QTL *BEC-A7b*. In general, fifteen QTLs had positive additive effects, showing that these alleles for higher phenotypic variation came from QY10. QTLs for different traits were clustered on A4, A7 and C4. Two robust QTLs for seed yield (*SYLB-A7*) and for seed weight (*SWLB-A7a*) co-localized on A7 with the favorable alleles contributed by QY10. Two QTL-by-environment interactions for PN and SN contributed 4.5% of the total phenotypic variation and were detected in A6 and C6 (Table 5).

**Table 5:** QTL-by-environment interactions identified in the BQDH population.

Trait	Chr.	Interval	Position	A	AE1	AE2	PVE%(A)	PVE%(AE)
LBPN	A6	CNU400-CNU325a	28.8	9.0235***	10.2712**		2.89	2.41
NBPN	C6	BoGMS1497-06Au-4	15.3	−8.9574***	−6.4949*	3.38	1.27	
LBSN	C6	BoGMS1497-06Au-4	12.3	−1.3858***	0.7099*	7.01	2.09	
BEC	C3	BoGMS0576-CB10427	65.9	−0.0407***	−0.0716***	0.0395*	2.45	4.95

Note:

PNLB, pot number under low B condition; PNNB, pot number under normal B condition; SNLB, seed number under low B condition; BEC, B efficiency coefficient.

Significance:

* *P*<0.05;

** <*P*<0.01;

*** *P*<0.001.

PVE: phenotypic variation explained.

Thirty-five putative QTLs were detected in eleven linkage groups under the NB condition (Table 4). Six QTLs were detected across three years and explained 5.10–23.26% of the phenotypic variation, and eleven QTLs were detected across two years and explained 4.75–16.98% of the phenotypic variation. Fifteen QTLs showed a positive effect, suggesting that these alleles for the advantageous phenotype were contributed by QY10. Two QTLs for pod number (*PNNB-C9*) and seed weight (*SWNB-C9*), as well as one QTL for branch number (*BNNB-C9b*), were clustered on C9. One QTL-by-environment interaction for PN explained 1.27% of the total phenotypic variation and was detected on C6 (Table 5).

Five putative QTLs for the BEC were detected on the A2, A7, C3 and C8 linkage groups and explained 6.8–11.44% of the phenotypic variation (Table 4). No QTL for the BEC was detected across two or three years, but seven QTLs for the BEC overlapped with QTLs for other tested traits under the LB condition. For example, *BEC-C8* co-localized with *SWLB-C8* on C8, contributing 7.33% of the phenotypic variation. One QTL-by-environment interaction for the BEC on C3 explained 4.95% of the total phenotypic variation (Table 5). Taken together, these imply that environment has a considerable effect on the performance of traits.

### Detection of epistatic interactions in the TNDH and BQDH populations

In total, ten epistatic interactions were detected for the SY under LB condition and the BEC in the TNDH population. These interactions contributed to 5.44%–14.38% of the phenotypic variation (Table 6). Eight of these interactions exhibited a negative effect, indicating that recombinant allele combinations could improve seed yield and the BEC. Two and three epistatic interactions were detected for the SY under the LB condition and the BEC in 2004 and contributed to 15.77% and 28.94% of the phenotypic variation, respectively. Two and three epistatic interactions were detected for the SY under the LB condition and the BEC in 2005 and explained 15.39% and 20.34% of the total phenotypic variation, respectively.

**Table 6:** Epistatic interactions for seed yield at low B condition (SYLB) and boron efficient coefficient (BEC) in the TN DH population of 2004 and 2005 field trials.

Trait	Chr-Int *i*	Markers	Chr-Int *j*	Markers	LOD	*Ai*	*R^2^(Ai)*	*Aj*	*R^2^(Aj)*	*AAij*	*R^2^(AAij)*
**2004**											
SYLB	A2-25	**S02M08-1-180/S08M15-170**	A9-49	CNU263/P13M10-265	4.72	−0.22	0.8	−0.08	0.1	−0.74	8.96
	A5-36	P10M6-190/sR9477	C8-16	CB10092/CB10028	4.46	−0.02	0.01	0.21	0.72	−0.64	6.81
BEC	A3-51	Na14G02/IGF0568c′	A9-19	pW235/CB10022	3.25	0.05	2.07	−0.01	0.04	0.08	5.28
	A4-14	E6HM40-160/CNU246	C7-4	sNRH63/Na10C01b	3.96	0.02	0.38	−0.03	0.97	−0.1	9.28
	C1-27	S08M15-85/IGF3141e	C3-19	SA27/sN2032	4.99	0.03	0.59	0.02	0.34	−0.13	14.38
**2005**											
SYLB	A4-10	IGF5193a/sN13034	A8-4	Na12B05a/CNU208	4.54	0.09	0.16	0.09	0.18	−0.62	8.2
	A9-37	pX150/S15M04-2-150	C1-14	E7HM40-590/S13M08-1-380	3.79	−0.15	0.5	0.14	0.41	0.58	7.19
BEC	A4-10	IGF5193a/sN13034	A10-16	AP1a/E5HM40-205	6.53	0.01	0.23	0	0.02	−0.08	8.33
	A5-28	IGF3165a/WG2E2	A7-18	sR7223/sNRA59	4.73	0	0.02	0	0	−0.07	6.57
	A9-48	pW123aH/CNU263	C2-14	SA12a/P5M5-1000	3.82	−0.02	0.77	0	0	−0.06	5.44

Note:

LOD score calculated by QTLmapper 2.0 at P≤0.005 level of probability.

*A*: The estimates of additive effect for testing point *i*; *j* and additive×additive epistasis *ij*.

R^2^: Proportion of phenotypic variation explained by the marker genotypes at the locus *i* or *j* and between the two testing points *i* and *j*.

Markers with bold means that the locus was located in QTL interval.

Thirty-two epistatic interactions involving 50 loci covering the whole genome, except A8, were detected for six measured traits under both B conditions, with the BEC contributing 0.53–14.26% of the phenotypic variation in the BQDH population (Table S3).

For SY, 10 epistatic interactions involving 19 loci were detected under the LB and NB conditions explaining 52.65% of the total phenotypic variation. One locus with an additive effect under the NB condition was involved in the epistatic interaction. For the BEC, 2 epistatic interactions involving 4 loci explained 7.1% of the total phenotypic variation with a positive effect. An interaction with the environment accounted for 6.23% of total phenotypic variation. For PN, one epistatic interaction involving a locus with an additive effect was detected explaining 1.62% of the phenotypic variation under the NB condition. For SN, a total of seven epistatic interactions involving 13 loci were detected under both conditions contributing 26.37% of the total phenotypic variation. Three loci with additive effects under the NB condition were involved in epistatic interactions. For BN, one epistatic interaction involving two loci with additive effect under the NB condition was found. Only one interaction with the environment contributed 1.74% of the phenotypic variation. For SW, six epistatic interactions involving eleven loci under the LB and the NB conditions were detected, and these interactions contributed 19.18% of the phenotypic variation. Six loci with additive effects were involved in epistatic interactions. For PH, five epistatic interactions involving nine loci explained 15.79% of the total phenotypic variation under the LB and NB conditions. Two loci with additive effects were involved. These results suggest that additive and additive-additive effects simultaneously control phenotypic variation under both B conditions. The additive effect was a major factor because the cumulative contribution from significant additive effects (4.15%–23.16%) was higher than the contribution from epistatic effects (0.53%–14.38%) for an individual trait.

### Associations of QTL with functional genes by *in silico* mapping

Twenty synteny blocks (A genome: 13, C genome: 7) and 123 insertion fragment islands (A genome: 77, C genome: 46) were identified between *Arabidopsis* pseudochromosomes and BQDH genetic linkage groups by the *in silico* mapping approach (Table S1). In total, 159 orthologous genes in *Arabidopsis* were mapped to the confidence intervals of 70 putative QTLs, corresponding to 30 blocks. Among the 159 genes, 23 were involved in B metabolism, and 136 were involved in yield-related trait control (Table S4). The 23 genes involved in B metabolism may be candidate genes underlying the QTLs specifically expressed under the LB condition. For example, *BOR7*, a member of the anion transporter family and homolog of *BOR1*, was located in the confidence intervals of *SYLB-A2a (b)* in the BQDH population and in the confidence intervals of *BEC-A2* and *SYLB-A2b* in the TNDH population (data not shown). Thus, *BOR7* is a potential candidate gene for B efficiency.

## Discussion

High yield is the most important goal in modern crop production, and high yield during abiotic stress, such as nutrient deficiency, represents an important area of study. The nutrient efficiency of a genotype is defined as the ability to produce higher yields in soils with limited nutrient supplies [36], [37]. We previously defined the BEC, which is the ratio of the yield under low B condition to that under normal B conditions, as B efficiency or the tolerance of a genotype to B deficiency [21]. Using the BEC as a screening index, B-efficient *B. napus* cultivars and *Arabidopsis* ecotypes were screened [38]. However, the BEC involving yield is a complex trait in plants regardless of the growth condition or environmental stress. In the present study, two DH populations were employed to identify QTLs for SY, SW, SN, PN, BN and PH under the LB and the NB conditions, and the BECs from two-year and three-year field trials were evaluated to determine the B efficiency of *B. napus*. Numerous QTLs for the seven traits were detected for both B conditions. These QTLs, especially those expressed in the LB condition, are valuable for dissecting the mechanism of B efficiency.

### Abundant variation during different B conditions

Phenotypic investigations showed abundant genetic variation in seven yield and yield-related traits between the two pairs of parents and between the two B conditions for the same genotype (Table 1, 2). In this study, the CV was calculated for both B conditions in the two populations, and a range from 11.5% (PHNB) to 114.1% (SYLB) throughout both B conditions was observed in the TNDH and BQDH populations. These traits were highly variable, especially the SY under the LB condition and the BEC in the two populations. In addition, there was considerable transgressive segregation for most of the traits under both B conditions. These results imply that yield and yield-related traits under the LB condition are highly variable and can be improved genetically.

Classical genetics assumed that the correlation among different traits is due to the tight linkage of genes affecting different traits or to genes with pleiotropic effects [39]. In this study, seed yield showed the highest correlation with the other measured traits (Table S2), and a high percentage (82%) of QTLs for SY co-localized with QTLs for other yield-related traits. On average, a QTL for SY involved 2 QTLs for yield-related traits under both B conditions, and a QTL for SY involved 1.4 QTLs for yield-related traits under the LB condition (Fig. S2). These results create a flexible approach for identifying the genetic basis of co-localized QTLs for SY under LB condition (i.e. B efficiency) by estimating which QTL of the yield-related trait(s) is the most probable locus for having linked genes or genes with pleiotropic effects on seed yield. Shi et al. (2009) facilitated the cloning of *qSY.A2-2*, a major QTL for seed yield, based on the hypothesis that *qFT.A2-4* controlled flowering time, which was considered as an indicator QTL [40]. The successful cloning of QTL for yield in rice [24] was facilitated by the indicator QTL for biomass yield.

### B efficiency QTLs and the genetic basis of B efficiency

In this study, the BEC was defined as the ability of a genotype to produce a high yield during B deficiency. Thus, the BEC was tightly associated with the SY under the LB condition. In the TNDH population, four putative QTLs for seed yield under the LB condition, *SYLB-A2a* and *SYLB-A2b* on A2, *SYLB-A6* on A6 and *SYLB-C4* on C4, and four putative QTLs for the BEC, *BEC-A2* on A2, *BEC-A4* on A4, *BEC-A6* on A6 and *BEC-C3* on C3, were identified. Among them, *BEC-A6*, *BEC-C3* and *SYLB-A2b* contributed 10.2%–18.5% of the phenotypic variation. In the BQDH population, seven putative QTLs for SY on A2, A7, C3, C4 and C7 under the LB condition, as well as seven putative QTLs for the BEC on A2, A7, C3 and C8, were identified. Among them, *BEC-C3*, *BEC-A7b* and *SYLB-A7* contributed 11.24%–14.25% of the phenotypic variation. These results suggest that B efficiency is regulated by a number of QTLs but is mainly controlled by the major QTLs, with higher than 10% of the PVE. Additionally, *SYLB-A6* and *BEC-A2* co-localized with *BEC-A6* and *SYLB-A2b*, respectively, in the TNDH population. *BEC-A2*and *BEC-C3* co-localized with *SYLB-A2* and *SYLB-C3*, respectively, in the BQDH population. This co-localization indicates that there is a close genetic associations between SYLB and the BEC and suggests that a single gene with pleiotropic effects or two linked genes can create tolerance to low B conditions.

**Figure 3:**
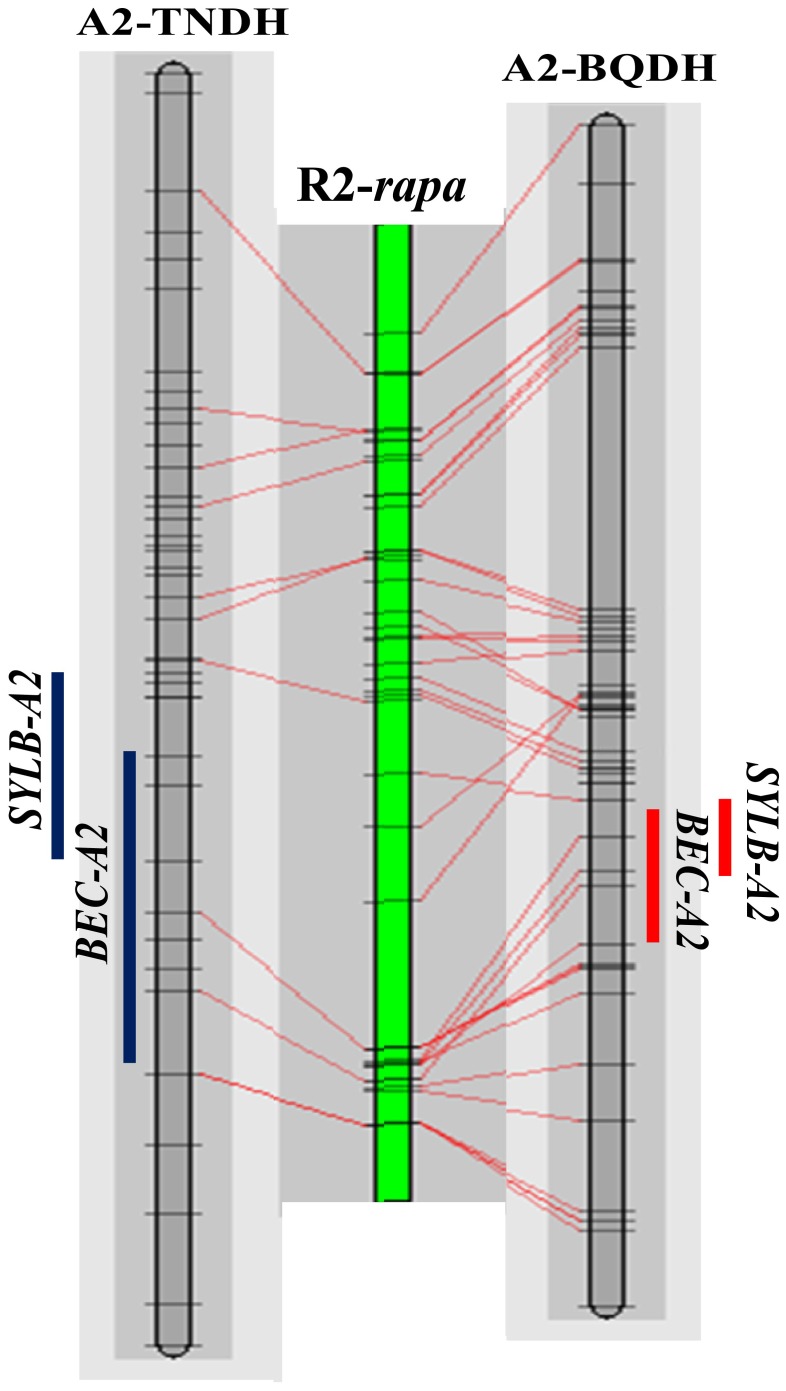
Co-location comparison of QTL interval for seed yield under low B (SYLB) and B efficiency coefficient (BEC) on A2 between the BQDH and the TNDH genetic maps by comparing with R2 of *B. rapa* genome. R2-*rapa* represented R2 chromosome in *B. rapa*.

**Figure 4:**
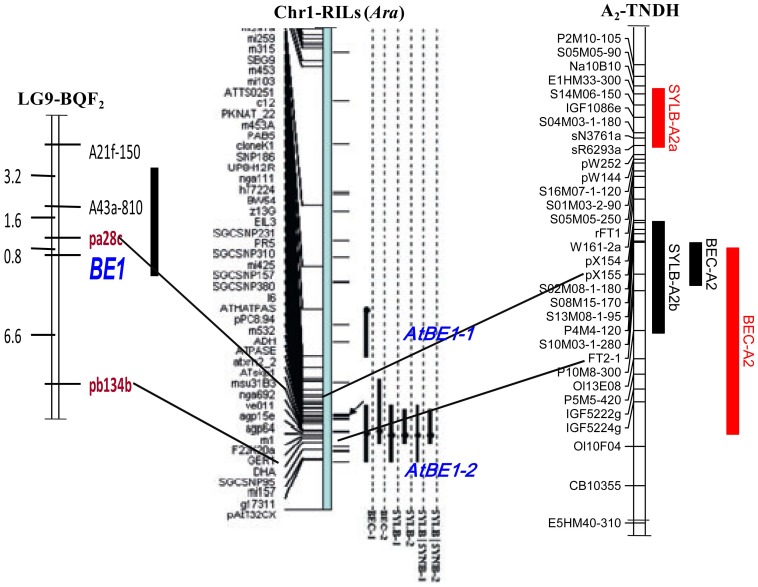
QTLs for seed yield at low B condition (SYLB) and B efficiency coefficient (BEC) in A2 linkage group of the TNDH population compared to B efficiency loci in LG9 linkage group of the BQ F_2_ population and *Arabidopsis* Chromosome 1. Black and red blocks means QTLs detected in the TNDH population in 2004 and 2005, respectively.

A comparative analysis between the TNDH and BQDH populations and R2 of the *B. rapa* genome validated the loci for the BEC and SY under the LB condition at A2 (Fig. 3). Previous studies have reported some loci for B efficiency in *B. napus* and *Arabidopsis*. Xu et al. (2001) mapped a major B efficiency QTL (*BE1*) to LG9 (LG9 was equivalent to recognized A2 linkage group) using an F_2_ population in *B. napus* [21]. Further, the *BE1* region mapped to an interval of 110.8–117.2 cM in length on *Arabidopsis* chromosome 1 using comparative mapping of two flanking RFLP markers (PB134-3∼PA28) [41]. Interestingly, the *BE1*-aligned region in *Arabidopsis* overlapped with the *Arabidopsis* B efficiency QTL *AtBE1-2* [38]. In this study, *BEC-A2* and *SYLB-A2b* in the TNDH population mapped to *AtBE1-2* in *Arabidopsis* by comparative mapping (Fig. 4). Therefore, we speculate that the genomic region on A2 acts as the major locus conferring B efficiency in *B. napus* and that this could be beneficial for fine mapping *BE1* in *B. napus*. Additionally, the QTLs *SYLB-A6* in TNDH and *SYLB-A7* in BQDH detected throughout the two seasons could be considered robust loci for fine mapping.

Several QTLs identified in other *B. napus* genetic populations were projected onto the BQDH genetic map based on the alignment analysis for common molecular markers between different genetic maps using the map projection function of BioMercator 2.1 software [42]. A total of 29 QTLs for six traits (SY, PH, BN, PN, SN and SW) [27], [29], [40], [43] were projected onto the BQDH population (Fig. S1). Among them, 2 QTLs were co-localized with QTLs identified under the NB condition (QTLs for BN on C6), 18 QTLs were co-localized with QTLs identified under the LB condition (such as 3 QTLs for PN on A7 and 2 QTLs for SN on A4), and 9 QTLs were co-localized with QTLs identified under both B conditions (such as 2 QTLs for SY on A7 and 2 QTLs for SW on C6). These results imply that common genetic factors could exist for some traits in different genetic backgrounds and that environments and some genetic factors may be associated with B efficiency.

Epistatic interactions are considered an important genetic mechanism for regulating phenotypic variation [44], [28]. In the present study, a number of epistatic interactions for yield and yield-related traits were identified. Interestingly, one locus (S02M08-1-180/S08M15-170) conferring the major QTL (*SYLB-A2b*) was associated with epistatic interactions, suggesting that epistatic interactions play important roles in controlling B efficiency in *B. napus*. Liu et al. (2009) identified 74 epistatic interactions regulating shoot mineral concentrations under different B conditions at the seedling stage in *B. napus* [45]. Previous studies successfully identified 45 differentially expressed proteins under B-limited condition at the seedling stage using the B-efficient cultivar QY10 [46]. The differentially expressed proteins covered eight metabolic pathways including antioxidant and detoxification, defense, signaling and regulation, carbohydrate and energy metabolism, amino acid and fatty acid metabolism and transport. These results suggest that a number of genes and interactions among associated genes could be involved in B efficiency and that they should not be neglected in MAS.

### B-related genes

Based on the speculated genomic region on A2 in *B. napus*, five candidate genes located in the equivalent region on R2 in *B. rapa* were screened (Table 7). The five candidate genes function as small molecule transporters. The homologous genes in *Arabidopsis*, AT5G27350 and AT5G27360, function as a superfamily of monosaccharide transporters [47]. AT5G28470 functions as a major facilitating protein and is associated with pollen tube growth and development [48]. AtNIP5;1 is a B transport channel protein [13].

**Table 7:** Candidate genes in *B.rapa* in the homologous region of the intervals of *SYLB-A2* and *BEC-A2* for B efficiency in *B. napus*.

Gene in *B.rapa*	Start	Stop	Functional annotation	Homologous gene in *Arabidopsis*	Gene in *AtBE1-2*
Bra020592	24377992	24383065	auxiliary transport protein activity;	AT5G27360	AT1G08930
Bra020593	24358466	24363733	transport accessory protein activity;	AT5G27350	
Bra020595	24340611	24343166	small molecule transport;	AT5G27350	
			solute:solute exchange;		
			small-molecule carrier or transporter;		
			cellular component.		
Bra020609	24233008	24236011	cellular component;	AT5G28470	AT1G08930
			oligopeptide transport;		
			small-molecule carrier or transporter.		
Bra033181	17297210	17300039	small-molecule carrier or transporter	AtNIP5;1	AtNIP5;1

In order to identify whether the homologous genes in *B. napus* localized to the A2 region for B efficiency, twelve pairs of gene-based simple sequence repeat (GB-SSR) primers were developed for four of the five candidate genes. The 9 and 8 GB-SSR markers that correspond to 10 and 8 loci were polymorphic in the BQDH and TNDH populations, respectively (Table 8). Moreover, the 10 and 8 loci were integrated into the expected intervals of the BQDH and TNDH genetic maps, respectively. Further, the latest results of QTL mapping for the BQDH population using an improved BQDH genetic map suggest that the loci Bra020595-1, Bra020595-2 and Bra020592-1a are located in the SYLB-A2 and BEC-A2 intervals. These results indicate that the Bra020592 and Bra020595 genes in *B. napus* are candidate genes for B efficiency.

**Table 8:** Sequence information for the polymorphic GB-SSR primers developed from the four candidate genes in *B. rapa*.

Locus	Repeat motif	Repeat start position (bp)	Primer sequence (5′-3′)	Expected size (bp)
Bra020592-1	(TA)_12_	24379844	GTGGCTGAACATCGGAAGAT	199
			CCTGCTTGGTACATCCATCA	
Bra020592-2	(AT)_10_	24382294	AGCGTCTGGGATGTGTTTGT	205
			TTGCATTTTCTGCTCGTACC	
Bra020592-3	(TA)_10_	24382840	GTTTGAATGGAGCACTCAAGG	202
			CCGGAACGAGAAACCAAATA	
Bra020593-1	(AT)_9_	24359107	CAAGTGTGGTCTTGTGGGAAT	196
			GTACGTTCGCATTCGGATACT	
Bra020593-2	(TA)_11_	24363010	GGCTCGCCTTCACATTACA	257
			TGCAAGATACAGCTGCCAAG	
Bra020595-1	(CT)_12_	24333492	CCTATTTGAGTTCCTTAAGCGATG	221
			GCTCCAAAAGCCCTTCTTCT	
Bra020595-2	(TC)_12_	24333517	CCCACTATCACAAAAACATAGCTC	205
			ACAGCCTGAAGCGTCACTTT	
Bra020595-3	(AT)_9_	24355967	GGTTGCATTGATGGCAAATAG	204
			GGCAAAGCATATGACAAAGC	
Bra020609-1	(A)_14_	24235380	TGGTACAAAAACCCCATGCT	174
			GGGCTAAAACCTGTTTTGGATT	

The comparative genome mapping analysis between *B. napus* and *Arabidopsis* revealed a common ancestor for the two species [49], [50]. It would be beneficial to resolve the genetic mechanisms of the complex *B. napus* genome using genetic information from the model plant *Arabidopsis*. Based on *in silico* mapping, some important genes involved in different biological processes in *Arabidopsis* were mapped to the target QTL intervals in *B. napus*. These QTLs were for flowering time [33], shoot mineral concentrations [45], seed yield and yield-related traits [40], [43], and P-efficiency traits [51]. In this study, a total of 44 genes involved in B uptake and transport [8], [13], [14], [15], [52] or genes induced by B-limited stress [46], [53] were used for the *in silico* mapping of the BQDH population. Twenty-three homologous genes were mapped to QTL intervals (Table S4), including B transporter family genes, B channel genes, biosynthetic process genes, KDOP synthase-related genes, cell structure-related genes, transport-related genes, abiotic stress-related genes, other genes induced by B deficiency, and transcription factors expressed during low B levels. For example, *NIP2;1*, a member of the NOD26-like intrinsic protein family, was localized to the interval of *SYLB-C3* and *BEC-C3*, explaining 7.19% and 11.44% of the phenotypic variation, respectively. Long et al (2007) identified *BnFLC10* as a potential candidate gene that controls flowering time by *in silico* mapping [33]. This finding suggests that it is possible to accelerate the process of cloning genes by *in silico* mapping. However, further fine mapping and analysis of near isogenic lines or association mapping will be needed to confirm the involvement of potential candidate genes.

A total of 35 pairs of gene-based marker (GBM) primers were developed for *Arabidopsis* B transporter and channel genes, tobacco cell wall pectin glucuronosyltransferase gene and for genes induced by B deficiency. Nine GBMs were integrated into the BQDH genetic map, and four of them were located in QTL intervals. Interestingly, two GBMs developed for the *Arabidopsis* B transporter genes were associated with putative QTLs detected under LB condition. ATBOR1-BrS3a, developed from *AtBOR1* [8], was mapped to the confidence interval of *SWLB-A1b* on A1, and NIP5;1-Bn4b developed from *NIP5;1* [13], was mapped to the confidence interval of *SWLB-C6* on C6. Two GBMs (NIP5;1-Br3a and NIP5;1-Br3b) were associated with four epistatic interactions, three of which were for SYLB and the BEC (Table S3). These results could be beneficial for identifying and cloning genes and could provide potential markers for MAS to produce B-efficient rapeseed cultivars.

## Materials and Methods

### Plant materials

Two double haploid populations TNDH and BQDH were employed in this study for mapping QTLs for yield and yield-related traits under different B conditions. The TNDH population was developed from a cross between Tapidor and Ningyou7 (NY7) [34]. Using the TNDH population, a genetic linkage map was constructed with 621 markers, including restriction fragment length polymorphisms (RFLPs), simple sequence repeats (SSRs) and single nucleotide polymorphisms (SNPs). The map covered 2060 cM on 19 chromosomes with an average interval between two adjacent markers of 3.3 cM [33]. The BQDH population, comprising 200 DH lines, was developed from one F_1_ progeny derived from a cross between QY10 (B-efficient) and Bakow (B-inefficient) [21] via the microspore culture technique.

### Field trials and traits investigation

For the TNDH population, a two-year field trial was conducted at our laboratory's boron fertilizer experiment base, which has sandy paddy soil, in Qichun county, Hubei Province (N 115°45′ E 30°19′), during the 2003–2004 and 2004–2005 crop seasons. No specific permits were required for the field trial. The average hot-water-soluble B in the plough layer soil before fertilization was 0.069 mg kg^−1^. In both trials, two B treatments were employed: the LB treatment, 1.5 kg ha^−1^ borax, and the NB treatment, 15 kg ha^−1^ borax. The NB treatment was the control, and the application of N-, P-, K-containing fertilizers for each treatment was according to the following nutrient rates: 180 kg N ha^−1^, 90 kg P_2_O_5_ ha^−1^, and 150 kg K_2_O ha^−1^. K as potassium chloride and P as ordinary superphosphate were applied as base fertilizers, and N as urea was divided into 120 kg before transplanting and 60 kg at the bolting stage. The seeds of the 202 DH lines, together with the two parents, were firstly sown in seedbed in the middle of September, and then the uniform seedlings were transplanted to B-treated field plots 30 days later. The plants were harvested at the beginning of the following May. The planting was conducted in a complete randomized block design with three replicates. Every block for a line contained two rows and the interval between adjacent rows was 25.6 cm, and 10 plants were planted in a row with an interval of 18 cm between adjacent plants. The seeds were sown by hand, and the field management followed standard agricultural practice. In each replicate, ten representative individuals of each block were harvested at physiological maturity. Seed yield was investigated for all the lines, and then the BEC for each DH line was calculated as the ratio of mean values of seed yield in three replicates under low B level to that under normal B level. Seed yield was recorded as the average seed dry weight of the harvested individuals.

For the BQDH population, a three-year field trial was carried out at our laboratory's boron fertilizer experiment base, which has sandy paddy soil, in Qichun county, Hubei Province (N 115°45′ E 30°19′), during the 2008–2009, 2009–2010 and 2010–2011 crop seasons. No specific permits were required for the field trial. The average hot-water-soluble B in the plough layer soil before fertilization was 0.09∼0.11 mg kg^−1^, and two B treatments were employed in the three trials. For the first trial, the LB treatment was 1.0 kg ha^−1^ of boric acid, which was sprayed as fertilizer before the bolting stage. For the second trial, no B fertilizer was applied throughout the season. For the third trial, 0.75 kg ha^−1^ of borax was applied as the base fertilizer. The NB treatment was the control, and the application of N-, P- and K-containing fertilizers, the trial block design and the agronomical management were the same as TNDH population field trial.

Six traits, including seed yield (SY), plant height (PH), branch number (BN), pod number (PN), seed number (SN) and seed weight (SW), were measured. One derived trait, the BEC, was calculated as the ratio of the seed yield under low B levels to that under normal B levels. The six traits were measured according to the methods described in [40].

### Analysis of polymorphic loci

Genomic DNA was extracted from plant leaf tissues by the cetyltrimethylammonium bromide (CTAB) method [54]. The genotypes of the BQDH lines were analyzed using simple sequence repeat (SSR), sequence-related amplified polymorphism (SRAP), and gene-based markers (GBMs) for the construction of a genetic linkage map.

Primer sequences of the SSR markers were obtained from various sources: UK prefixed by OL and Na (http://www.brasscia.bbsrc.ac.uk/BrassicaDB), Australia prefixed by sA (http://www.hornbill.cspp.latrobe.edu.au), Canada prefixed by sR and sN (http://www.brassica.agr.gc.ca/index_e.shtml), Japan prefixed by BRMS [55], and France prefixed by BRAS, CB and MR [56]. A total of 171 *B.rapa* BAC sequences and/or BAC-end sequences (BES) primers [57], 698 SSR primers designated as BnGMS [58] and 1398 SSR primers designated as BoGMS [59] were used. Additionally, due to the release of the genome sequence of *B.rapa* [60], we developed 50 SSR primers designated as BeA2ssr according to the A2 sequences to improve the A2linkage group in the BQDH genetic linkage map.

The analysis of the SRAP markers has been previously described [61], and the polymorphic primer pairs were named by combining the names of the forward and reverse primers (e.g., em5me24).

The gene-based markers (GBMs) were functional genes related to B transporters and channel proteins in *Arabidopsis* (http://www.arabidopsis.org/). The primers were designed based on the conserved sequences between the homologous genes from *Arabidopsis* and *Brassica*. The GBMs were named by using the suffix Bn or Br and the gene name in *Arabidopsis*, for example AtNIP5;1-Bn. The analysis of GBM followed the protocol described by [57].

All PCR products were separated by PAGE and stained with AgNO_3_. If a primer pair showed more than one polymorphic loci, the different loci were distinguished by small letters after the name of the marker. For example, the primer pair CB10079 generated two polymorphic loci, which were named CB10079a and CB10079b and were distinguished by the product size in increasing order.

### Linkage analysis and map construction

Linkage analysis and map construction were performed using the JoinMap software Version 4.0 [62]. The threshold for goodness-of-fit was set to ≤5.0 with a recombination frequency of <0.4 and a minimum logarithm of odds score of 1.0. Markers with a χ^2^ value of >3.0 were excluded in all genetic groups. Recombination frequencies were converted to centimorgans (cM) using Kosambi's method for map distance calculation [63].

### QTLs, interaction detection and *in silico* mapping

QTL detection was carried out by the composite interval method (CIM) [64], using WinQTLcart 2.5 software [65]. CIM was performed using Model 6 after scanning the genetic map and estimating the likelihood of a QTL and its corresponding effect every 1 cM. The number of control markers and the window size were set to 5 and 10 cM, respectively. For each trait, the threshold for detection of a significant QTL (P<0.05) was estimated by 1,000 permutations [66]. The estimated additive effect and the percentage of phenotypic variation explained by each putative QTL were obtained using the software with the CIM model. QTL support intervals were determined by 2-LOD intervals surrounding the QTL peak. When QTLs for the same trait during the same B treatment across two or three years had overlapping support intervals, they were assumed to be consistent.

Epistatic interactions and environmental interactions were detected using the software program QTLmapper 2.0 (http://www.cab.zju.edu.cn/ics/faculty/zhujun.htm) (for the TNDH population) and the software program QTLNetwork2.0 (for the BQDH population) [67], which are all based on mixed linear model approaches [68]. The 1D search for QTL×environment interval effects was carried out with a 10 cM testing window, a 1 cM walking speed, and a 5 cM window size. Both 1D and 2D genome scans were conducted with *p*<0.05 significance threshold based on 1,000 permutations.

The *in silico* mapping of the BQDH population was carried out according to the method described in [49]. Genes were identified by comparative mapping between the *B. napus* linkage groups and the *A. thaliana* genome in each syntenic block of *A. thaliana* and then associated with each putative QTL. If the position of an aligned gene(s) was located in the support interval of a QTL, the orthologous gene(s) was considered to be associated with the target QTL.

### Statistical analysis

Statistical analysis for all traits was conducted using SAS8.1 (SAS Institute, Cary, NC, USA). Histograms and normality tests (Pearson chi-square test) were used to describe the variation of the phenotypic traits. The Pearson's phenotypic correlation coefficients among seven traits across all environments were calculated to examine their phenotypic association using the SAS PROC CORR. ANOVA was conducted using the SAS general linear model (GLM) procedure.

## Supporting Information

Figure S1 QTL projection from other populations onto the BQDH genetic linkage map, via a map projection using BioMercator 2.1 software (Arcade et al. 2004) based on common markers.
(DOCX)

Figure S2 Chromosomal locations of putative QTLs for yield and yield-related traits in *Brassica napus* BQDH population.
(DOCX)

Table S1 The BQDH genetic linkage map and its syntenic segmental alignment with the *Arabidopsis* genome.
(DOCX)

Table S2 Pearson's correlation analysis between traits in TNDH (A) and BQDH (B) populations.
(DOCX)

Table S3 Epistatic interactions for six yield related traits under both B conditions and BEC in BQDH population of *Brasscia napus*.
(DOCX)

Table S4 Orthologous genes associated with yield and yield-associated QTLs detected in BQDH population by *in-silico* mapping between *A.thaliana* and *B.napus*.
(DOCX)
