# Supplementary material for: Dissecting Quantitative Trait Loci for Boron Efficiency across Multiple Environments in Brassica napus
Source: PLoS One. 2012 Sep 24;7(9):e45215. doi: 10.1371/journal.pone.0045215 (PMC3454432; doi:10.1371/journal.pone.0045215)
Supplement: Figure S1 — QTL projection from other populations onto the BQDH genetic linkage map, via a map projection using BioMercator 2.1 software (Arcade et al. 2004) based on common markers. (DOCX) [file pone.0045215.s001.docx]

**Fig S1** QTL projection from another population onto BQDH genetic map, via a map-projection using BioMercator 2.1 software (Arcade et al. 2004) based on common markers. The QTLs projected onto BQDH genetic map were labeled with dark blue, and the QTLs in other colours were detected in the present study. The dashed lines denote common markers between the BQDH genetic map and other genetic maps in which the QTL projection was processed.
