# Supplementary material for: Dissecting Quantitative Trait Loci for Boron Efficiency across Multiple Environments in Brassica napus
Source: PLoS One. 2012 Sep 24;7(9):e45215. doi: 10.1371/journal.pone.0045215 (PMC3454432; doi:10.1371/journal.pone.0045215)
Supplement: Figure S2 — Chromosomal locations of putative QTLs for yield and yield-related traits in Brassica napus BQDH population. (DOCX) [file pone.0045215.s002.docx]

**Fig S2** Chromosomal locations of putative QTLs for yield and yield-related traits in *Brassica napus* BQDH population. The length of the vertical coloured line to the right of the chromosomes indicates two-LOD support intervals. The peak position of QTL was shown by horizontal bars on the vertical coloured line. The coloured bars to the left of the chromosomes represent different preudochromosomes of *A. thaliana* that have been aligned to the linkage map of *B. napus* according to the 24 *Arabidopsis* genomic blocks identified by Schranz et al. (2006). Two gene-based markers located in the QTL intervals under low B condition on A1 and C6 were shown to the right of the linkage group.
