# Supplementary material for: Dissecting Quantitative Trait Loci for Boron Efficiency across Multiple Environments in Brassica napus
Source: PLoS One. 2012 Sep 24;7(9):e45215. doi: 10.1371/journal.pone.0045215 (PMC3454432; doi:10.1371/journal.pone.0045215)
Supplement: Table S1 — The BQDH genetic linkage map and its syntenic segmental alignment with the Arabidopsis genome. (DOCX) [file pone.0045215.s003.docx]

**Table S1** The BQDH genetic linkage map and its syntenic segmental alignment with the *Arabidopsis* genome

| **LG** | **Marker name** | **Marker type** | **Position (cM)** | **Syntenic interval in Arabidopsis genome** | | | | **Conserved block in *B. napus* genome** | |
| --- | --- | --- | --- | --- | --- | --- | --- | --- | --- |
|  |  |  |  | **Homologous locus** | **Block *** | **Left (bp)** | **Right (bp)** | **Left (cM)** | **Right(cM)** |
| A1 | HAU123-6 | SSR | 0.00 |  |  |  |  |  |  |
| A1 | CB10081 | SSR | 5.39 |  |  |  |  |  |  |
| A1 | niab108 | SSR | 13.53 | At4g36080.1 | U | 18,098,559 | 9,195,617 | 11.93 | 63.21 |
| A1 | niab071 | SSR | 18.37 | At4g34540.1 |  |  |  |  |  |
| A1 | BP2607-Br1 | GBM | 20.50 |  |  |  |  |  |  |
| A1 | B036M17 | SSR | 21.99 | AT4G34220 |  |  |  |  |  |
| A1 | CNU139 | SSR | 24.62 | At4g33460.1 |  |  |  |  |  |
| A1 | CB10097 | SSR | 27.84 |  |  |  |  |  |  |
| A1 | B035P03-1 | SSR | 32.96 |  |  |  |  |  |  |
| A1 | BoGMS1162 | SSR | 33.58 | AT4G32150.1 |  |  |  |  |  |
| A1 | BnGMS676 | SSR | 35.75 | AT4G31460.1 |  |  |  |  |  |
| A1 | ea10 | SSR | 38.28 |  |  |  |  |  |  |
| A1 | BoGMS0789 | SSR | 46.51 |  |  |  |  |  |  |
| A1 | CB10099 | SSR | 49.85 |  |  |  |  |  |  |
| A1 | B2-S4-1 |  | 56.33 |  |  |  |  |  |  |
| A1 | BoGMS1565b | SSR | 59.68 | AT4G21550.1 |  |  |  |  |  |
| A1 | CNU107 | SSR | 69.11 | At3g20530.1 | F | 6,166,053 | 8,167,935 | 67.55 | 70.67 |
| A1 | AS0 | SSR | 78.13 |  |  |  |  |  |  |
| A1 | ATBOR1-BrS3a | GBM | 79.69 | AT2G47160.2 | J | 18,357,478 | 19,566,533 | 78.13 | 80.01 |
| A1 | BoGMS1224 | SSR | 80.48 |  |  |  |  |  |  |
| A1 | BRMS011 | SSR | 81.46 |  |  |  |  |  |  |
| A1 | BRMS031 | SSR | 82.68 |  |  |  |  |  |  |
| A1 | H001C24 | SSR | 84.14 | At3g15080.1 | F | 4,074,223 | 6,076,531 | 82.58 | 85.70 |
| A1 | CB10159a | SSR | 88.24 |  |  |  |  |  |  |
| A1 | FITO514b | SSR | 97.16 |  |  |  |  |  |  |
| A1 | CB10597 | SSR | 100.81 |  |  |  |  |  |  |
| A1 | BRAS043 | SSR | 104.04 |  |  |  |  |  |  |
| A1 | Na14F11 | SSR | 111.58 | AT3G06270.1 | F | 896,137 | 2,898,034 | 110.02 | 113.14 |
| A1 | BRAS074c | SSR | 114.05 |  |  |  |  |  |  |
| A2 | sR94102a | SSR | 0.00 |  |  |  |  |  |  |
| A2 | H004D11 | SSR | 6.22 | At5g10170.1 | R | 2,187,278 | 6,827,155 | 4.66 | 15.59 |
| A2 | niab105 | SSR | 13.97 | At5g17680.1 |  |  |  |  |  |
| A2 | B071M18 | SSR | 14.03 | At5g17680.1 |  |  |  |  |  |
| A2 | B048M01-1 | SSR | 17.18 | At5g60390 | W | 24,308,246 | 23,952,433 | 17.15 | 18.82 |
| A2 | H007A03-1 | SSR | 18.66 | At5g59660.1 |  |  |  |  |  |
| A2 | BeA2ssr44 | SSR | 18.97 | AT5G02030.1 | R | 295,154 | 1,021,761 | 18.82 | 19.95 |
| A2 | BeA2ssr43b | SSR | 20.20 |  |  |  |  |  |  |
| A2 | B071K22 | SSR | 20.92 | At5g54490.1 | W | 21,515,910 | 22,262,997 | 19.95 | 21.11 |
| A2 | BnGMS566 | SSR | 21.31 | AT1G32750.1 | B | 11,722,407 | 11,980,285 | 21.11 | 21.50 |
| A2 | B001N22 | SSR | 21.69 | At5g54670.1 | W | 22,103,178 | 22,618,240 | 21.50 | 22.30 |
| A2 | BeA2ssr06 | SSR | 22.90 | AT5G03545.1 | R | 507,826 | 1,895,400 | 22.30 | 24.46 |
| A2 | BoGMS0987 | SSR | 49.68 |  |  |  |  |  |  |
| A2 | BeA2ssr09 | SSR | 50.43 |  |  |  |  |  |  |
| A2 | BeA2ssr11 | SSR | 50.99 |  |  |  |  |  |  |
| A2 | BoGMS0892 | SSR | 51.51 |  |  |  |  |  |  |
| A2 | BoGMS0965a | SSR | 52.45 |  |  |  |  |  |  |
| A2 | BoGMS1010 | SSR | 52.90 |  |  |  |  |  |  |
| A2 | BnGMS332 | SSR | 53.87 |  |  |  |  |  |  |
| A2 | em5me28d | SSR | 57.46 |  |  |  |  |  |  |
| A2 | BoGMS0659 | SSR | 58.17 |  |  |  |  |  |  |
| A2 | BoGMS0500 | SSR | 58.38 |  |  |  |  |  |  |
| A2 | BeA2ssr29a | SSR | 58.65 | AT5G66760.1 | X | 25,653,658 | 26,904,430 | 57.09 | 59.04 |
| A2 | BoGMS0972 | SSR | 59.28 |  |  |  |  |  |  |
| A2 | BoGMS0660 | SSR | 59.62 |  |  |  |  |  |  |
| A2 | BeA2ssr14 | SSR | 59.81 | AT5G02900.1 | R | 179,439 | 676,849 | 59.04 | 59.81 |
| A2 | BeA2ssr13 | SSR | 59.82 | AT3G29160.3 | L | 11,127,426 | 11,169,963 | 59.81 | 59.87 |
| A2 | B070J11-2 | SSR | 59.93 | At1g65295.1 | E | 24,217,094 | 25,255,966 | 59.87 | 61.49 |
| A2 | BRAS011a | SSR | 60.54 |  |  |  |  |  |  |
| A2 | BeA2ssr17 | SSR | 64.12 | AT3G06170.1 | F | 867,415 | 2,394,108 | 62.56 | 64.93 |
| A2 | BnGMS635 | SSR | 65.16 |  |  |  |  |  |  |
| A2 | BeA2ssr18 | SSR | 65.75 | AT5G48150.2 | V | 18,998,095 | 19,532,698 | 64.93 | 65.77 |
| A2 | BoGMS1250a | SSR | 65.78 | AT3G10290.1 | F | 3,175,403 | 3,355,908 | 65.77 | 66.04 |
| A2 | BoGMS1472b | SSR | 66.31 | AT3G28200.1 | L | 10,347,510 | 10,827,006 | 66.04 | 66.79 |
| A2 | BnGMS103 | SSR | 67.27 | AT4G28080.1 | U | 13,640,763 | 14,554,960 | 66.79 | 68.21 |
| A2 | BRAS042 | SSR | 67.38 |  |  |  |  |  |  |
| A2 | BeA2ssr23 | SSR | 69.14 | AT5G23350.1 | Q | 7,261,133 | 8,859,387 | 68.21 | 70.70 |
| A2 | FITO372 | SSR | 72.78 |  |  |  |  |  |  |
| A2 | OL12B03b | SSR | 76.42 | AT5G49300.1 |  |  |  |  |  |
| A2 | H092G20b | SSR | 77.98 | AT5G27460.1 | Q | 8,693,262 | 10,694,840 | 76.42 | 79.54 |
| A2 | OL12B03a | SSR | 83.88 | AT5G49300.1 |  |  |  |  |  |
| A2 | BeA2ssr38 | SSR | 85.77 | AT5G60800.1 | X | 25,460,887 | 23,438,271 | 84.21 | 87.75 |
| A2 | BeA2ssr39 | SSR | 86.19 | AT5G60760.1 |  |  |  |  |  |
| A2 | BnGMS629 | SSR | 86.27 |  |  |  |  |  |  |
| A2 | CB10540 | SSR | 88.99 |  |  |  |  |  |  |
| A2 | B059L17 | SSR | 96.15 | At5g26830.1 | Q | 8,437,300 | 10,441,782 | 94.59 | 97.71 |
| A2 | BRMS026 | SSR | 101.84 |  |  |  |  |  |  |
| A2 | B086G22a | SSR | 111.18 | At5g23450.1 | Q | 6,904,756 | 8,519,792 | 109.62 | 112.14 |
| A2 | OL10F04 | SSR | 112.23 |  |  |  |  |  |  |
| A2 | B086G22b | SSR | 113.09 | At5g23450.1 | Q | 7,294,516 | 8,909,552 | 112.14 | 114.65 |
| A2 | B021J09 | SSR | 120.77 | At5g66840.1 | X | 25,710,148 | 26,904,430 | 119.21 | 120.77 |
| A3 | B055N13-3 | SSR | 0.00 | At5g06220.1 | R | 880,193 | 2,885,644 | 0.00 | 14.88 |
| A3 | niab115a | SSR | 11.16 | At5g06220.1 |  |  |  |  |  |
| A3 | niab115b | SSR | 13.32 | At5g06220.1 |  |  |  |  |  |
| A3 | CB10079a | SSR | 16.37 |  |  |  |  |  |  |
| A3 | HBr112a | SSR | 22.97 | At5g16460.1 | R | 4,377,088 | 6,035,496 | 21.41 | 23.99 |
| A3 | Na12E02a | SSR | 25.02 | AT2G33880.1 | J | 13,684,470 | 15,343,778 | 23.99 | 26.58 |
| A3 | CB10036c | SSR | 25.70 |  |  |  |  |  |  |
| A3 | MR123a | SSR | 31.27 |  |  |  |  |  |  |
| A3 | sR6688 | SSR | 32.27 |  |  |  |  |  |  |
| A3 | BnGMS79 | SSR | 33.25 | AT5G56200.1 | W | 21,747,768 | 23,749,249 | 31.69 | 34.81 |
| A3 | CNU253 | SSR | 36.58 | At2g34680.1 | J | 13,215,983 | 19,566,533 | 34.82 | 56.31 |
| A3 | Na12E02b | SSR | 38.00 | AT2G33880.1 |  |  |  |  |  |
| A3 | S012M03-1 | SSR | 39.29 | At2g34680.1 |  |  |  |  |  |
| A3 | HAU93-3 | SSR | 41.29 | At2g36380.1 |  |  |  |  |  |
| A3 | CNU288 | SSR | 45.83 | At2g39800.1 |  |  |  |  |  |
| A3 | ATBOR1-Bn4 | GBM | 55.94 | AT2G47160.2 |  |  |  |  |  |
| A3 | Na10B01 | SSR | 58.12 | AT2G26580.1 | I | 10,303,590 | 12,306,850 | 56.31 | 59.68 |
| A3 | HBr052 | SSR | 62.66 | AT4G02650.1 | O | 156,453 | 2,158,653 | 61.10 | 64.22 |
| A3 | BoGMS1117 | SSR | 64.70 |  |  |  |  |  |  |
| A3 | BoGMS0644 | SSR | 65.42 |  |  |  |  |  |  |
| A3 | CNU098 | SSR | 67.98 | At3g01290.1 | F | 8,964 | 1,213,517 | 67.86 | 69.69 |
| A3 | HAU122-2 | SSR | 69.13 | At3g03550.1 |  |  |  |  |  |
| A3 | BoGMS1146 | SSR | 70.18 |  |  |  |  |  |  |
| A3 | ATBOR1-BrS3b | GBM | 70.26 | AT2G47160.2 | J | 18,995,558 | 19,366,587 | 69.69 | 70.27 |
| A3 | FITO285 | SSR | 70.27 | AT3G05710.2 | F | 1,692,998 | 489,419 | 70.27 | 71.86 |
| A3 | BoGMS1422b | SSR | 70.30 | AT3G05220.2 |  |  |  |  |  |
| A3 | BnGMS417b | SSR | 77.79 |  |  |  |  |  |  |
| A3 | BRAS102a | SSR | 88.47 |  |  |  |  |  |  |
| A3 | MR12 | SSR | 92.68 |  |  |  |  |  |  |
| A3 | CB10415 | SSR | 96.06 |  |  |  |  |  |  |
| A3 | BnGMS584b | SSR | 100.75 |  |  |  |  |  |  |
| A3 | CNU370 | SSR | 108.36 | At3g49870.1 | M | 17,503,393 | 18,540,051 | 106.80 | 108.41 |
| A3 | B043L22 | SSR | 109.33 |  |  |  |  |  |  |
| A3 | CNU223b | SSR | 111.85 | At3g51730.1 | N | 18,946,655 | 19,926,070 | 111.45 | 112.98 |
| A3 | CNU409 | SSR | 114.12 | At4g15440.1 | T | 7,835,494 | 9,137,215 | 112.98 | 114.58 |
| A3 | CNU435 | SSR | 116.14 | At4g16620.1 | U | 9,195,617 | 18,098,559 | 115.43 | 140.72 |
| A3 | CNU492 | SSR | 121.82 | AT4G19930 |  |  |  |  |  |
| A3 | BnGMS523 | SSR | 125.84 |  |  |  |  |  |  |
| A3 | BoGMS1251 | SSR | 127.14 |  |  |  |  |  |  |
| A3 | HAU61 | SSR | 135.24 | At4g28140.1 |  |  |  |  |  |
| A3 | BoGMS0582b | SSR | 136.70 |  |  |  |  |  |  |
| A3 | BoGMS1539 | SSR | 137.14 |  |  |  |  |  |  |
| A3 | BnGMS291 | SSR | 138.42 |  |  |  |  |  |  |
| A3 | H009I12-1b | SSR | 139.13 | At4g36080.1 |  |  |  |  |  |
| A3 | BnGMS392b | SSR | 139.28 |  |  |  |  |  |  |
| A3 | BoGMS0408 | SSR | 139.49 |  |  |  |  |  |  |
| A3 | BoGMS0661 | SSR | 139.71 |  |  |  |  |  |  |
| A3 | BnGMS416b | SSR | 140.36 |  |  |  |  |  |  |
| A3 | BRAS050 | SSR | 141.11 |  |  |  |  |  |  |
| A4 | CB10493a | SSR | 0.00 |  |  |  |  |  |  |
| A4 | BoGMS0252b | SSR | 7.65 | AT2G42080.1 | J | 19,566,533 | 13,215,983 | 4.51 | 35.01 |
| A4 | BoGMS1408 | SSR | 11.59 | AT2G41020.1 |  |  |  |  |  |
| A4 | CNU256 | SSR | 15.49 | At2g38280.1 |  |  |  |  |  |
| A4 | H004D08 | SSR | 17.86 | At2g38280.1 |  |  |  |  |  |
| A4 | BoGMS1067 | SSR | 33.99 | AT2G32710.1 |  |  |  |  |  |
| A4 | BoGMS0965b | SSR | 41.26 |  |  |  |  |  |  |
| A4 | sN11516 | SSR | 43.44 |  |  |  |  |  |  |
| A4 | BoGMS0798 | SSR | 45.78 | AT2G28360.1 | I | 9,075,399 | 12,424,462 | 45.32 | 53.16 |
| A4 | S006L21-1 | SSR | 48.70 | At2g27920.1 |  |  |  |  |  |
| A4 | B014N06 | SSR | 48.87 | At2g27730.1 |  |  |  |  |  |
| A4 | BnGMS628 | SSR | 52.97 |  |  |  |  |  |  |
| A4 | BoGMS0266 | SSR | 53.22 |  |  |  |  |  |  |
| A4 | BoGMS1041 | SSR | 58.62 |  |  |  |  |  |  |
| A4 | BnGMS115 | SSR | 60.39 | AT5G50230.1 | W | 20,054,564 | 21,450,881 | 59.77 | 61.95 |
| A4 | B022O03 | SSR | 61.72 | At4g39950.1 |  |  |  |  |  |
| A4 | CB10347 | SSR | 64.10 |  |  |  |  |  |  |
| A4 | sN0464b | SSR | 68.05 |  |  |  |  |  |  |
| A5 | sNRD03 | SSR | 0.00 |  |  |  |  |  |  |
| A5 | ATBOR1-Br7 | GBM | 7.97 | AT2G47160.2 | J | 19,566,533 | 13,215,983 | 7.65 | 51.72 |
| A5 | BRAS072a | SSR | 10.46 |  |  |  |  |  |  |
| A5 | BRMS034 | SSR | 15.60 |  |  |  |  |  |  |
| A5 | CNU286b | SSR | 24.30 | At2g45490.1 |  |  |  |  |  |
| A5 | CNU257 | SSR | 30.46 | At2g39090.1 |  |  |  |  |  |
| A5 | BRAS063 | SSR | 35.53 |  |  |  |  |  |  |
| A5 | B023F24b | SSR | 38.07 | At2g39090.1 |  |  |  |  |  |
| A5 | CNU344 | SSR | 44.95 | At2g35920.1 |  |  |  |  |  |
| A5 | sN12353 | SSR | 47.06 |  |  |  |  |  |  |
| A5 | BoGMS0033 | SSR | 47.84 | AT2G36800.1 |  |  |  |  |  |
| A5 | BoGMS0766 | SSR | 48.81 | AT2G36130.1 |  |  |  |  |  |
| A5 | MR119 | SSR | 59.69 |  |  |  |  |  |  |
| A5 | BnGMS662 | SSR | 65.13 |  |  |  |  |  |  |
| A5 | BoGMS1422a | SSR | 66.88 | AT3G05220.2 | F | 489,419 | 2,274,354 | 65.32 | 68.10 |
| A5 | BnGMS294 | SSR | 68.36 |  |  |  |  |  |  |
| A5 | BoGMS0287 | SSR | 68.85 |  |  |  |  |  |  |
| A5 | Bac-26 | SSR | 69.33 | AT1G49360.1 | C | 17,484,191 | 19,285,482 | 68.10 | 69.83 |
| A5 | BoGMS1562 | SSR | 69.70 | AT1G51760.1 |  |  |  |  |  |
| A5 | BoGMS0231 | SSR | 69.94 |  |  |  |  |  |  |
| A5 | Bac-23 | SSR | 69.96 | AT1G49360.1 | C | 18,183,391 | 18,271,048 | 69.83 | 69.96 |
| A5 | B026E21-1 | SSR | 69.97 | AT1G31880.1 | B | 11,443,890 | 11,463,629 | 69.96 | 69.99 |
| A5 | BnGMS667 | SSR | 69.99 |  |  |  |  |  |  |
| A5 | CNU029 | SSR | 70.01 | At1g48130.1 | C | 17,770,731 | 17,818,356 | 69.99 | 70.06 |
| A5 | CNU398 | SSR | 70.11 | At3g22400.1 | F | 7,893,926 | 8,177,884 | 70.06 | 70.50 |
| A5 | FITO357 | SSR | 70.24 | AT3G22630.1 |  |  |  |  |  |
| A5 | CNU325b | SSR | 70.35 |  |  |  |  |  |  |
| A5 | BoGMS1472a | SSR | 70.76 | AT3G28200.1 | L | 10,351,030 | 10,875,326 | 70.50 | 71.32 |
| A5 | Bac-19 | SSR | 70.98 |  |  |  |  |  |  |
| A5 | H026E14a | SSR | 71.88 | AT3G17790.1 | F | 8,964 | 9,272,627 | 71.49 | 113.24 |
| A5 | FITO513 | SSR | 72.61 | AT3G19950.1 |  |  |  |  |  |
| A5 | H026E14b | SSR | 73.29 | AT3G17790.1 |  |  |  |  |  |
| A5 | CB10080 | SSR | 77.39 |  |  |  |  |  |  |
| A5 | H001C24-1 | SSR | 90.62 | At3g15080.1 |  |  |  |  |  |
| A5 | niab082 | SSR | 93.22 | At3g15080.1 |  |  |  |  |  |
| A5 | BoGMS0581 | SSR | 98.39 | AT3G12680.1 |  |  |  |  |  |
| A5 | B087P06 | SSR | 108.17 | At3g10470.1 |  |  |  |  |  |
| A5 | BnGMS265 | SSR | 109.10 |  |  |  |  |  |  |
| A5 | BnGMS230 | SSR | 115.15 | AT5G67290.1 | X | 25,848,148 | 26,904,430 | 113.59 | 115.24 |
| A5 | sR9477 | SSR | 119.48 |  |  |  |  |  |  |
| A5 | CNU362 | SSR | 124.52 | At3g08590.1 | F | 1,608,477 | 3,611,424 | 122.96 | 124.52 |
| A6 | FITO314c | SSR | 0.00 | AT5G44340.1 | V | 18,861,101 | 7,801,814 | 0.00 | 3.90 |
| A6 | H089G09 | SSR | 2.34 | AT5G44200.1 |  |  |  |  |  |
| A6 | BoGMS0746a | SSR | 6.65 |  |  |  |  |  |  |
| A6 | CNU400 | SSR | 24.80 | At3g26720.1 | L | 9,460,658 | 10,824,423 | 24.24 | 26.36 |
| A6 | CNU325a | SSR | 35.67 |  |  |  |  |  |  |
| A6 | BnGMS331 | SSR | 55.48 |  |  |  |  |  |  |
| A6 | BnGMS531 | SSR | 59.12 | AT5G64813.1 | X | 24,910,279 | 26,904,430 | 57.56 | 60.26 |
| A6 | AT6 | SSR | 60.57 |  |  |  |  |  |  |
| A6 | FITO316 | SSR | 61.40 | AT3G49080.1 | M | 17,463,594 | 18,540,051 | 60.26 | 61.93 |
| A6 | AT2a | SSR | 61.66 |  |  |  |  |  |  |
| A6 | CNU149 | SSR | 63.80 | At1g20930.1 | B | 6,886,879 | 8,294,735 | 63.16 | 65.36 |
| A6 | BoGMS0950 | SSR | 69.95 |  |  |  |  |  |  |
| A6 | BnGMS366 | SSR | 73.98 |  |  |  |  |  |  |
| A6 | BRMS030 | SSR | 74.65 |  |  |  |  |  |  |
| A6 | BnGMS132 | SSR | 84.94 | AT4G23460.1 | U | 11,243,671 | 13,248,950 | 83.38 | 86.50 |
| A6 | sN2837a | SSR | 99.05 |  |  |  |  |  |  |
| A6 | sN2837b | SSR | 104.20 |  |  |  |  |  |  |
| A7 | HAU34-1 | SSR | 0.00 | At2g20290.1 | H | 7,750,356 | 9,069,172 | 0.00 | 0.48 |
| A7 | sR0282Ra | SSR | 15.00 |  |  |  |  |  |  |
| A7 | sR0282Rb | SSR | 15.55 |  |  |  |  |  |  |
| A7 | BoGMS1575 | SSR | 19.36 | AT1G25480.1 | B | 7,948,465 | 8,959,836 | 17.80 | 19.38 |
| A7 | BoGMS0531 | SSR | 19.39 | AT2G17700.1 | H | 7,676,428 | 8,689,436 | 19.38 | 20.95 |
| A7 | HAU49-4 | SSR | 29.71 | At1g25390.1 | B | 7,906,439 | 9,908,841 | 28.15 | 31.27 |
| A7 | BRMS040 | SSR | 35.20 |  |  |  |  |  |  |
| A7 | MD20 | SSR | 41.69 |  |  |  |  |  |  |
| A7 | BRAS023 | SSR | 45.16 |  |  |  |  |  |  |
| A7 | BRMS018 | SSR | 47.18 |  |  |  |  |  |  |
| A7 | BRMS005 | SSR | 56.47 |  |  |  |  |  |  |
| A7 | sR7223b | SSR | 67.71 |  |  |  |  |  |  |
| A7 | sR7223a | SSR | 68.75 |  |  |  |  |  |  |
| A7 | B084H08-1 | SSR | 70.30 | At3g56240.1 | N | 19,874,236 | 23,149,434 | 68.74 | 73.45 |
| A7 | B027O09 | SSR | 71.89 | At3g59920.1 |  |  |  |  |  |
| A7 | niab043 | SSR | 73.68 | AT1G80420.1 | E | 30,243,427 | 24,163,497 | 73.67 | 85.13 |
| A7 | BoGMS1530 | SSR | 75.06 |  |  |  |  |  |  |
| A7 | CNU167 | SSR | 76.15 | At1g71691.1 |  |  |  |  |  |
| A7 | niab030 | SSR | 77.90 | At1g72210.1 |  |  |  |  |  |
| A7 | CNU331 | SSR | 80.78 | At1g74300.1 |  |  |  |  |  |
| A7 | Nip5;1-Br3a | GBM | 98.15 | AT4G10380.1 | P | 6,958,670 | 5,551,312 | 96.78 | 98.97 |
| A7 | BoGMS0721c | SSR | 99.79 | AT5G10750.1 | R | 2,874,791 | 4,400,156 | 98.97 | 99.79 |
| A8 | BoGMS0724 | SSR | 0.00 |  |  |  |  |  |  |
| A8 | BoGMS0774 | SSR | 22.40 | AT1G26610.1 | B | 8,193,350 | 10,195,280 | 20.84 | 23.96 |
| A8 | Ah7 | SSR | 22.77 |  |  |  |  |  |  |
| A8 | BoGMS0018 | SSR | 28.04 |  |  |  |  |  |  |
| A8 | AA8 | SSR | 29.00 |  |  |  |  |  |  |
| A8 | sR3688 | SSR | 31.88 | At4G38490 | U | 18,098,559 | 9,195,617 | 31.74 | 51.63 |
| A8 | niab090 | SSR | 41.29 | At4g26890.1 |  |  |  |  |  |
| A8 | BnGMS452 | SSR | 43.40 |  |  |  |  |  |  |
| A8 | HAU38-2 | SSR | 45.20 | At4g30180.1 |  |  |  |  |  |
| A8 | BnGMS492 | SSR | 48.70 |  |  |  |  |  |  |
| A8 | Bac-81 | SSR | 50.47 |  |  |  |  |  |  |
| A8 | FITO131 | SSR | 51.08 | AT4G17785.1 |  |  |  |  |  |
| A8 | S008P19-1 | SSR | 51.09 | At4g18040.1 |  |  |  |  |  |
| A8 | CNU489 | SSR | 52.41 | At4g14740.1 | T | 7,946,568 | 8,581,703 | 51.63 | 52.61 |
| A8 | CB10364 | SSR | 52.41 |  |  |  |  |  |  |
| A8 | B019A15-1 | SSR | 52.81 | At4g14740.1 | T | 8,322,607 | 9,137,215 | 52.61 | 53.88 |
| A8 | Bac-9 | SSR | 54.30 |  |  |  |  |  |  |
| A8 | BoGMS1069 | SSR | 58.41 | AT1G53750.1 | C | 19,065,714 | 21,017,677 | 56.85 | 58.41 |
| A9 | HBr132 | SSR | 0.00 | At1g04210.1 | A | 202,136 | 6,683,529 | 0.00 | 18.18 |
| A9 | HBr134 | SSR | 3.83 | At1g04210.1 |  |  |  |  |  |
| A9 | BoGMS1558 | SSR | 9.64 | AT1G10820.2 |  |  |  |  |  |
| A9 | sR5795d | SSR | 14.08 |  |  |  |  |  |  |
| A9 | HBr185 | SSR | 17.32 | AT1G18150.2 |  |  |  |  |  |
| A9 | H081N08-1 | SSR | 20.17 | At2g21330.1 | I | 8,135,232 | 10,370,283 | 18.18 | 23.88 |
| A9 | CNU008 | SSR | 20.71 | At2g21330.1 |  |  |  |  |  |
| A9 | HBr116 | SSR | 22.32 | At2g22000.1 |  |  |  |  |  |
| A9 | CNU372 | SSR | 48.06 | At3g53280.1 | N | 18,946,655 | 20,768,591 | 46.78 | 49.62 |
| A9 | niab046 | SSR | 55.22 | At1g26330.1 | B | 6,886,879 | 13,617,445 | 51.75 | 68.51 |
| A9 | CNU076 | SSR | 58.40 |  |  |  |  |  |  |
| A9 | niab047 | SSR | 59.58 | At1g26500.1 |  |  |  |  |  |
| A9 | HBr002 | SSR | 62.01 | At1g27400.1 |  |  |  |  |  |
| A9 | MR83.1 | SSR | 63.25 |  |  |  |  |  |  |
| A9 | FITO520 | SSR | 64.63 |  |  |  |  |  |  |
| A9 | S010I09-C4-1a | SSR | 64.97 | At1g27450.1 |  |  |  |  |  |
| A9 | HBr196 | SSR | 65.19 | At1g27400.1 |  |  |  |  |  |
| A9 | niab039 | SSR | 65.81 | At1g27450.1 |  |  |  |  |  |
| A9 | B017D04 | SSR | 66.40 | At1g28440.1 |  |  |  |  |  |
| A9 | S010I09-C0-33 | SSR | 66.63 | At1g27450.1 |  |  |  |  |  |
| A9 | BoGMS0603 | SSR | 69.03 | AT4G11920.1 | P | 6,828,564 | 7,234,495 | 68.51 | 69.14 |
| A9 | Na10G06 | SSR | 69.37 |  |  |  |  |  |  |
| A9 | B056G23 | SSR | 71.23 | At4g17110.1 | U | 9,195,617 | 10,563,990 | 70.57 | 72.71 |
| A9 | BnGMS124 | SSR | 74.19 | AT4G04850.2 | O | 1,504,184 | 2,525,278 | 72.71 | 74.29 |
| A9 | CB10029 | SSR | 77.56 |  |  |  |  |  |  |
| A9 | B034P04-3 | SSR | 81.45 | At5g46590.1 | V | 17,922,552 | 19,683,662 | 79.89 | 82.63 |
| A9 | sA5a | SSR | 85.70 |  |  |  |  |  |  |
| A9 | CB10103 | SSR | 93.49 |  |  |  |  |  |  |
| A9 | BnGMS493 | SSR | 96.54 | AT1G63295.1 | D | 22,474,069 | 23,667,906 | 94.98 | 96.84 |
| A9 | BRAS055 | SSR | 104.47 |  |  |  |  |  |  |
| A10 | OL10B11 | SSR | 0.00 |  |  |  |  |  |  |
| A10 | niab034 | SSR | 3.46 | At1g03020.1 | A | 202,136 | 6,683,529 | 2.69 | 11.81 |
| A10 | B030F10 | SSR | 5.89 | At1g03020.1 |  |  |  |  |  |
| A10 | BoGMS0949 | SSR | 7.38 | AT1G05170.2 |  |  |  |  |  |
| A10 | sORH13a | SSR | 9.13 |  |  |  |  |  |  |
| A10 | BnGMS9 | SSR | 9.16 | AT1G05490.1 |  |  |  |  |  |
| A10 | BRMS017 | SSR | 11.19 |  |  |  |  |  |  |
| A10 | BnGMS460 | SSR | 11.87 |  |  |  |  |  |  |
| A10 | BnGMS171 | SSR | 12.34 | AT5G52200.1 | W | 20,202,639 | 22,204,759 | 11.81 | 13.90 |
| A10 | BoGMS0197b | SSR | 13.27 |  |  |  |  |  |  |
| A10 | H034P05 | SSR | 18.17 | AT5G58960.1 | W | 22,805,717 | 24,308,246 | 16.61 | 18.95 |
| A10 | CB10524 | SSR | 26.91 |  |  |  |  |  |  |
| A10 | niab123 | SSR | 35.02 | At5g18060.1 | R | 7,293,597 | 97,893 | 25.85 | 52.26 |
| A10 | Bac-12 | SSR | 36.42 |  |  |  |  |  |  |
| A10 | FITO057 | SSR | 41.62 | AT5G15890.1 |  |  |  |  |  |
| A10 | BoGMS1593 | SSR | 44.98 | AT5G15260.1 |  |  |  |  |  |
| A10 | BoGMS1510a | SSR | 47.62 | AT5G14180.1 |  |  |  |  |  |
| A10 | BoGMS1510c | SSR | 48.01 | AT5G14180.1 |  |  |  |  |  |
| A10 | BnGMS385 | SSR | 50.56 | AT5G49435.1 |  |  |  |  |  |
| A10 | sN8474 | SSR | 60.85 |  |  |  |  |  |  |
| A10 | CB10079c | SSR | 68.90 |  |  |  |  |  |  |
| A10 | FITO481 | SSR | 75.82 | AT3G11510.1 | F | 2,623,457 | 4,624,940 | 74.26 | 77.38 |
| A10 | niab099 | SSR | 79.40 | At5g05800.1 | R | 743,087 | 2,745,246 | 77.84 | 79.40 |
| C1 | BoGMS1294 | SSR | 0.00 |  |  |  |  |  |  |
| C1 | CB10369b | SSR | 6.32 |  |  |  |  |  |  |
| C1 | CB10369a | SSR | 8.51 |  |  |  |  |  |  |
| C1 | BoGMS1565a | SSR | 16.03 | AT4G21550.1 | U | 9,195,617 | 18,098,559 | 12.49 | 29.26 |
| C1 | BoGMS0292 | SSR | 20.18 | AT4G24390.1 |  |  |  |  |  |
| C1 | BoGMS0794 | SSR | 20.74 | AT4G24430.1 |  |  |  |  |  |
| C1 | CB10277 | SSR | 24.15 |  |  |  |  |  |  |
| C1 | BoGMS1568 | SSR | 26.04 |  |  |  |  |  |  |
| C1 | BnGMS271 | SSR | 32.59 | AT3G49850.1 | M | 17,489,034 | 18,540,051 | 31.03 | 32.66 |
| C1 | BoGMS0840 | SSR | 34.86 |  |  |  |  |  |  |
| C1 | BoGMS556 | SSR | 35.39 | AT3G23430.1 | F | 7,387,508 | 9,272,627 | 33.83 | 36.02 |
| C1 | BnGMS299 | SSR | 36.18 |  |  |  |  |  |  |
| C1 | BoGMS0658 | SSR | 36.75 | AT2G27430.1 | I | 10,729,826 | 12,424,462 | 36.02 | 37.83 |
| C1 | BoGMS0721a | SSR | 48.76 | AT5G10750.1 | R | 2,398,951 | 4,400,156 | 47.20 | 50.32 |
| C1 | BnGMS268 | SSR | 50.26 |  |  |  |  |  |  |
| C1 | BnGMS394 | SSR | 52.65 | AT5G14420.2 | R | 3,648,111 | 4,971,629 | 51.09 | 53.15 |
| C1 | Na10H03 | SSR | 53.65 | AT3G12130.1 | F | 3,543,832 | 5,980,241 | 53.15 | 57.61 |
| C1 | BnGMS370 | SSR | 56.05 | AT3G14820.1 |  |  |  |  |  |
| C1 | BRAS074a | SSR | 64.96 |  |  |  |  |  |  |
| C2 | sR12095a | SSR | 0.00 |  |  |  |  |  |  |
| C2 | B2-S2-1 |  | 3.50 |  |  |  |  |  |  |
| C2 | BoGMS1290 | SSR | 6.17 | AT5G07600.1 | R | 1,404,574 | 6,932,963 | 4.61 | 12.05 |
| C2 | HBr024 | SSR | 10.49 | At5g18580.1 |  |  |  |  |  |
| C2 | BeA2ssr05 | SSR | 12.39 |  |  |  |  |  |  |
| C2 | BoGMS1123 | SSR | 12.85 | AT5G56190.2 | W | 24,308,246 | 20,054,564 | 12.82 | 16.69 |
| C2 | BeA2ssr02 | SSR | 12.90 |  |  |  |  |  |  |
| C2 | BeA2ssr43a | SSR | 12.90 |  |  |  |  |  |  |
| C2 | BeA2ssr45 | SSR | 13.20 |  |  |  |  |  |  |
| C2 | B048M01-2 | SSR | 13.57 | At5g60390 |  |  |  |  |  |
| C2 | Na14H11 | SSR | 14.50 | AT5G55190.1 |  |  |  |  |  |
| C2 | BRAS083 | SSR | 15.32 |  |  |  |  |  |  |
| C2 | B060E11-1 | SSR | 17.63 | At5g20490.1 | R | 5,926,625 | 7,293,597 | 16.69 | 18.19 |
| C2 | sR94102b | SSR | 23.31 |  |  |  |  |  |  |
| C3 | CB10079b | SSR | 0.00 |  |  |  |  |  |  |
| C3 | CB10358 | SSR | 15.94 |  |  |  |  |  |  |
| C3 | BoGMS0358 | SSR | 25.56 | AT5G03455.1 | R | 97,893 | 7,293,597 | 24.37 | 38.17 |
| C3 | BoGMS1332 | SSR | 25.75 |  |  |  |  |  |  |
| C3 | niab115c | SSR | 26.32 | At5g06220.1 |  |  |  |  |  |
| C3 | BoGMS0953 | SSR | 30.20 | AT5G05210.2 |  |  |  |  |  |
| C3 | BoGMS1288 | SSR | 36.32 | AT5G18420.3 |  |  |  |  |  |
| C3 | CB10036a | SSR | 39.92 |  |  |  |  |  |  |
| C3 | CB10036b | SSR | 40.49 |  |  |  |  |  |  |
| C3 | BoGMS1193 | SSR | 46.47 | AT5G58640.2 | W | 24,308,246 | 22,596,272 | 45.52 | 49.23 |
| C3 | BoGMS0678 | SSR | 47.67 | AT5G58375.1 |  |  |  |  |  |
| C3 | MR123b | SSR | 54.16 |  |  |  |  |  |  |
| C3 | HBr025 | SSR | 62.65 | At2g31410.1 | J | 13,215,983 | 19,566,533 | 62.37 | 85.24 |
| C3 | BoGMS0576 | SSR | 64.91 | AT2G31840.1 |  |  |  |  |  |
| C3 | CB10427 | SSR | 68.87 |  |  |  |  |  |  |
| C3 | BoGMS0693 | SSR | 73.09 |  |  |  |  |  |  |
| C3 | OL10E05 | SSR | 75.59 | AT2G37860.3 |  |  |  |  |  |
| C3 | BoGMS0577 | SSR | 76.44 | AT2G38400.2 |  |  |  |  |  |
| C3 | OL10B04 | SSR | 77.57 |  |  |  |  |  |  |
| C3 | BoGMS1587 | SSR | 80.36 | AT2G39360.1 |  |  |  |  |  |
| C3 | BoGMS0348 | SSR | 84.61 |  |  |  |  |  |  |
| C3 | sNRA56 | SSR | 89.48 |  |  |  |  |  |  |
| C3 | NIP5;1-Bn4a | GBM | 93.22 | AT4G10380.1 | P | 5,551,312 | 7,234,495 | 91.85 | 94.47 |
| C3 | BnGMS283 | SSR | 95.61 | AT4G04955.1 | O | 2,525,278 | 1,476,489 | 95.61 | 98.37 |
| C3 | BnGMS461 | SSR | 96.81 | AT4G04890.1 |  |  |  |  |  |
| C3 | BnGMS584a | SSR | 138.79 | AT2G05990.2 | G | 1,869,874 | 3,325,146 | 138.09 | 140.35 |
| C3 | BoGMS0728 | SSR | 141.75 |  |  |  |  |  |  |
| C3 | BoGMS0508 | SSR | 143.51 |  |  |  |  |  |  |
| C3 | BoGMS0570 | SSR | 156.76 |  |  |  |  |  |  |
| C3 | BnGMS289 | SSR | 165.50 |  |  |  |  |  |  |
| C3 | CNU208 | SSR | 169.53 | AT1G67980.2 | E | 24,487,714 | 26,489,158 | 167.97 | 171.09 |
| C3 | BoGMS0746b | SSR | 177.19 |  |  |  |  |  |  |
| C3 | FITO314b | SSR | 181.79 | AT5G44340.1 | V | 17,254,393 | 18,861,101 | 180.85 | 183.35 |
| C3 | FITO094 | SSR | 194.71 | AT4G23713.1 | U | 11,352,956 | 13,353,131 | 193.15 | 194.71 |
| C4 | CB10320 | SSR | 0.00 |  |  |  |  |  |  |
| C4 | BoGMS1357 | SSR | 1.65 |  |  |  |  |  |  |
| C4 | BRAS072b | SSR | 7.13 |  |  |  |  |  |  |
| C4 | BoGMS0252a | SSR | 14.82 | AT2G42080.1 | J | 13,215,983 | 19,566,533 | 8.06 | 33.88 |
| C4 | BnGMS275 | SSR | 15.15 |  |  |  |  |  |  |
| C4 | CNU286a | SSR | 28.30 | At2g45490.1 |  |  |  |  |  |
| C4 | BoGMS1219 | SSR | 32.75 | AT2G45740.3 |  |  |  |  |  |
| C4 | B023F24a | SSR | 37.80 | At2g39090.1 | J | 17,324,378 | 15,225,235 | 36.24 | 41.39 |
| C4 | BnGMS479 | SSR | 40.00 | AT2G38500.1 |  |  |  |  |  |
| C4 | BnGMS259 | SSR | 42.79 | AT1G63350.1 | D | 22,602,775 | 23,667,906 | 41.39 | 42.96 |
| C4 | sA55 | SSR | 44.55 |  |  |  |  |  |  |
| C4 | MR229 | SSR | 49.24 |  |  |  |  |  |  |
| C4 | B050K06 | SSR | 50.69 | At2g35920.1 | J | 14,082,676 | 16,087,891 | 49.13 | 52.25 |
| C4 | em14me20 | SRAP | 58.04 |  |  |  |  |  |  |
| C4 | BnGMS266 | SSR | 59.04 |  |  |  |  |  |  |
| C4 | em3me24b | SRAP | 59.57 |  |  |  |  |  |  |
| C4 | BoGMS1025 | SSR | 60.00 |  |  |  |  |  |  |
| C4 | em18me15a | SRAP | 60.84 |  |  |  |  |  |  |
| C4 | FITO514a | SSR | 61.32 |  |  |  |  |  |  |
| C4 | BoGMS1011 | SSR | 61.38 | AT5G19180.1 | R | 5,453,276 | 7,293,597 | 59.82 | 62.68 |
| C4 | BoGMS1049 | SSR | 61.38 |  |  |  |  |  |  |
| C4 | BoGMS0083 | SSR | 61.38 |  |  |  |  |  |  |
| C4 | em18me15b | SRAP | 62.43 |  |  |  |  |  |  |
| C4 | em15me7b | SRAP | 63.26 |  |  |  |  |  |  |
| C4 | BoGMS0326 | SSR | 67.53 | AT3G58190.1 | N | 22,549,558 | 18,946,655 | 65.97 | 72.41 |
| C4 | B087P03-1 | SSR | 71.01 | At3g53490.1 |  |  |  |  |  |
| C4 | sN0464a | SSR | 76.32 |  |  |  |  |  |  |
| C4 | BRAS021b | SSR | 81.97 |  |  |  |  |  |  |
| C4 | BRAS021a | SSR | 83.23 |  |  |  |  |  |  |
| C4 | BRAS003 | SSR | 84.77 |  |  |  |  |  |  |
| C4 | FITO210 | SSR | 91.84 |  |  |  |  |  |  |
| C4 | CNU250 | SSR | 107.98 | At2g32940.1 | J | 13,215,983 | 14,984,553 | 106.79 | 107.98 |
| C5 | sN13039 | SSR | 0.00 | AT1G03060.1 | A | 1,726,891 | 202,136 | 0.00 | 7.96 |
| C5 | BoGMS0382 | SSR | 7.85 | AT1G01770.1 |  |  |  |  |  |
| C5 | FITO88 | SSR | 8.39 |  |  |  |  |  |  |
| C5 | sORH13b | SSR | 24.59 |  |  |  |  |  |  |
| C5 | BoGMS0039 | SSR | 38.68 | AT1G09250.1 | A | 1,989,378 | 6,683,529 | 37.12 | 55.49 |
| C5 | BoGMS0319 | SSR | 54.25 | AT1G17210.1 |  |  |  |  |  |
| C5 | S010I09-C4-1c | SSR | 65.05 | At1g27450.1 | B | 8,531,826 | 10,534,101 | 63.49 | 66.61 |
| C5 | BnGMS352 | SSR | 66.46 |  |  |  |  |  |  |
| C5 | BnGMS433 | SSR | 69.33 |  |  |  |  |  |  |
| C5 | BoGMS1297 | SSR | 70.11 | AT1G48920.1 | C | 8,098,095 | 19,101,623 | 68.55 | 71.67 |
| C5 | sS2129 | SSR | 70.53 |  |  |  |  |  |  |
| C5 | S010I09-C4-1b | SSR | 86.53 | At1g27450.1 | B | 8,531,826 | 10,534,101 | 84.97 | 86.53 |
| C6 | BoGMS0721b | SSR | 0.00 |  |  |  |  |  |  |
| C6 | Nip5;1-Br3b | GBM | 0.00 | AT4G10380.1 | P | 5,551,312 | 7,234,495 | 0.00 | 1.25 |
| C6 | CNU053b | SSR | 4.07 | At1g77310.1 | E | 24,163,497 | 30,243,427 | 2.09 | 31.96 |
| C6 | BoGMS1497 | SSR | 5.31 | AT1G77480.1 |  |  |  |  |  |
| C6 | O6Au-4 | SSR | 22.20 |  |  |  |  |  |  |
| C6 | BoGMS0347b | SSR | 24.45 | AT1G67830.1 |  |  |  |  |  |
| C6 | BRMS036 | SSR | 26.75 |  |  |  |  |  |  |
| C6 | BoGMS0742 | SSR | 29.49 |  |  |  |  |  |  |
| C6 | HS-WA3 | SSR | 30.58 |  |  |  |  |  |  |
| C6 | BRAS116 | SSR | 31.50 |  |  |  |  |  |  |
| C6 | NIP5;1-Bn4b | GBM | 37.71 | AT4G10380.1 | P | 5,551,312 | 7,234,495 | 36.33 | 38.95 |
| C6 | MR133.1 | SSR | 40.11 |  |  |  |  |  |  |
| C6 | BoGMS0614 | SSR | 54.06 |  |  |  |  |  |  |
| C7 | BnGMS380 | SSR | 0.00 |  |  |  |  |  |  |
| C7 | sN0706 | SSR | 1.82 |  |  |  |  |  |  |
| C7 | BRAS019 | SSR | 3.69 |  |  |  |  |  |  |
| C7 | sNRH63b | SSR | 4.76 |  |  |  |  |  |  |
| C7 | sNRH63a | SSR | 5.58 |  |  |  |  |  |  |
| C7 | BoGMS0833 | SSR | 13.75 | AT1G26820.1 | B | 10,293,784 | 6,886,879 | 12.19 | 18.60 |
| C7 | BoGMS0381 | SSR | 17.39 | AT1G21840.1 |  |  |  |  |  |
| C7 | BoGMS0108 | SSR | 41.14 | AT2G01880.1 | K | 132,696 | 1,149,058 | 40.73 | 42.32 |
| C7 | BoGMS1453 | SSR | 46.86 | AT3G25905.1 | L | 9,460,658 | 10,486,771 | 46.82 | 48.42 |
| C7 | sA32 | SSR | 54.49 |  |  |  |  |  |  |
| C7 | BoGMS1585 | SSR | 67.02 | AT2G38470.1 | J | 15,108,361 | 17,110,766 | 65.46 | 68.58 |
| C7 | CNU223a | SSR | 81.35 | At3g51730.1 | N | 18,946,655 | 20,199,670 | 81.74 | 82.91 |
| C7 | CB10034 | SSR | 88.99 |  |  |  |  |  |  |
| C7 | BoGMS1013 | SSR | 94.29 |  |  |  |  |  |  |
| C7 | BoGMS1012 | SSR | 95.25 |  |  |  |  |  |  |
| C7 | BoGMS0582a | SSR | 110.12 | AT4G29100.1 | U | 13,341,000 | 15,344,930 | 108.56 | 111.68 |
| C7 | BnGMS392a | SSR | 124.06 |  |  |  |  |  |  |
| C7 | H009I12-1a | SSR | 125.02 | At4g36080.1 | U | 16,059,827 | 18,077,631 | 123.46 | 125.37 |
| C7 | BnGMS416a | SSR | 125.37 |  |  |  |  |  |  |
| C8 | CB10028b | SSR | 0.00 |  |  |  |  |  |  |
| C8 | H138P04-1 | SSR | 11.91 | At1g07280.1 | A | 202,136 | 6,683,529 | 10.19 | 21.97 |
| C8 | BoGMS0796a | SSR | 13.58 |  |  |  |  |  |  |
| C8 | HAU109 | SSR | 15.42 | At1g04680.1 |  |  |  |  |  |
| C8 | BnGMS302 | SSR | 20.15 |  |  |  |  |  |  |
| C8 | BoGMS0722 | SSR | 20.94 | AT1G13290.1 |  |  |  |  |  |
| C8 | H012G24 | SSR | 22.45 | At1g49290.1 | C | 17,927,506 | 19,237,292 | 21.97 | 24.01 |
| C8 | sR5795b | SSR | 23.40 |  |  |  |  |  |  |
| C8 | B025K04 | SSR | 29.97 | At1g63540.1 | D | 23,667,906 | 21,919,534 | 29.83 | 32.40 |
| C8 | BoGMS1078 | SSR | 30.84 | AT1G62020.1 |  |  |  |  |  |
| C8 | CNU356 | SSR | 33.51 | At2g22530.1 | I | 9,075,399 | 11,579,230 | 32.72 | 39.96 |
| C8 | BoGMS1027 | SSR | 38.40 | AT2G24830.1 |  |  |  |  |  |
| C8 | FITO508 | SSR | 44.51 | AT3G62200.1 | N | 22,023,437 | 23,235,283 | 42.95 | 44.84 |
| C8 | CB10373 | SSR | 50.97 |  |  |  |  |  |  |
| C8 | BoGMS1377 | SSR | 55.82 | AT3G57650.1 | N | 20,349,507 | 22,353,174 | 54.26 | 57.38 |
| C8 | BnGMS375 | SSR | 63.66 |  |  |  |  |  |  |
| C8 | BoGMS1460 | SSR | 80.34 |  |  |  |  |  |  |
| C8 | ew1 | SSR | 81.45 |  |  |  |  |  |  |
| C8 | BnGMS509 | SSR | 93.83 |  |  |  |  |  |  |
| C8 | BnGMS486 | SSR | 107.55 |  |  |  |  |  |  |
| C8 | BoGMS1474 | SSR | 108.43 |  |  |  |  |  |  |
| C8 | BnGMS336 | SSR | 108.95 |  |  |  |  |  |  |
| C8 | BoGMS1145 | SSR | 109.20 |  |  |  |  |  |  |
| C8 | BoGMS0302 | SSR | 109.81 |  |  |  |  |  |  |
| C8 | sA22 | SSR | 115.75 |  |  |  |  |  |  |
| C9 | HAU14-4 | SSR | 0.00 | At5g48370.1 | V | 18,615,749 | 19,683,662 | 0.00 | 0.10 |
| C9 | BnGMS679a | SSR | 5.06 | AT4G03520.2 | O | 562,505 | 2,525,278 | 3.50 | 6.56 |
| C9 | CB10064b | SSR | 8.13 |  |  |  |  |  |  |
| C9 | FITO516 | SSR | 30.41 | AT5G27650.1 | Q | 8,785,407 | 10,789,232 | 28.85 | 31.33 |
| C9 | HBr303b | SSR | 32.25 | At5g66460.1 | X | 25,555,621 | 26,904,430 | 31.33 | 32.79 |
| C9 | sN1988 | SSR | 33.89 |  |  |  |  |  |  |
| C9 | em5me28c | SRAP | 37.74 |  |  |  |  |  |  |
| C9 | BoGMS0143 | SSR | 39.57 |  |  |  |  |  |  |
| C9 | BoGMS1178 | SSR | 40.38 | AT1G56590.1 | D | 22,204,742 | 21,178,433 | 38.82 | 41.77 |
| C9 | HBr155 | SSR | 41.74 | At1g56570.1 |  |  |  |  |  |
| C9 | BnGMS213b | SSR | 43.95 |  |  |  |  |  |  |
| C9 | BoGMS1561 | SSR | 53.16 | AT2G01980.1 | K | 132,696 | 1,149,058 | 52.65 | 54.23 |
| C9 | sNRG42 | SSR | 58.19 |  |  |  |  |  |  |
| C9 | BnGMS351 | SSR | 60.63 |  |  |  |  |  |  |
| C9 | BoGMS0197a | SSR | 66.55 |  |  |  |  |  |  |
| C9 | BoGMS0594 | SSR | 71.29 |  |  |  |  |  |  |
| C9 | BoGMS0200 | SSR | 72.97 |  |  |  |  |  |  |
| C9 | BnGMS325 | SSR | 73.21 |  |  |  |  |  |  |
| C9 | CB10288 | SSR | 78.14 |  |  |  |  |  |  |
| C9 | sR12384la | SSR | 86.46 |  |  |  |  |  |  |

* Block definition for the Arabidopsis genome is according to Schranz et al. (2006) introduced. The limits of the conserved blocks in each linkage group

were defined according to the approach described by Long *et al.* (2007).
