# Supplementary material for: Dissecting Quantitative Trait Loci for Boron Efficiency across Multiple Environments in Brassica napus
Source: PLoS One. 2012 Sep 24;7(9):e45215. doi: 10.1371/journal.pone.0045215 (PMC3454432; doi:10.1371/journal.pone.0045215)
Supplement: Table S2 — Pearson's correlation analysis between traits in TNDH (A) and BQDH (B) populations. (DOCX) [file pone.0045215.s004.docx]

**Table S2** Pearson’s correlation analysis between traits in the TNDH and BQDH populations.

|  | **SYNB-1** | **SYLB-1** | **BEC-1** | **SYNB-2** | **SYLB-2** |
| --- | --- | --- | --- | --- | --- |
| **SYLB-1** | 0.61^***^ |  |  |  |  |
| **BEC-1** | -0.2 | 0.62^***^ |  |  |  |
| **SYNB-2** | 0.78^***^ | 0.44^***^ | -0.2 |  |  |
| **SYLB-2** | 0.45^***^ | 0.61^***^ | 0.26^*^ | 0.53^***^ |  |
| **BEC-2** | -0.07 | 0.32^**^ | 0.44^***^ | -0.19 | 0.66^***^ |

| **2009** | **SW** | **PH** | **SN** | **PN** | **BN** | **SY** | **BEC** |
| --- | --- | --- | --- | --- | --- | --- | --- |
| **SW** |  | -0.06 | 0.08 | 0.04 | -0.01 | 0.33*** | 0.16* |
| **PH** | -0.23** |  | 0.28*** | 0.18*** | 0.30*** | 0.33*** | 0.14 |
| **SN** | -0.24*** | 0.34*** |  | -0.07 | -0.01 | 0.61*** | 0.18* |
| **PN** | -0.10 | 0.45*** | 0.23** |  | 0.146 | 0.45*** | 0.36*** |
| **BN** | -0.31*** | 0.16* | 0.03 | 0.29*** |  | 0.11 | 0.04 |
| **SY** | 0.03 | 0.46*** | 0.73*** | 0.69*** | 0.09 |  | 0.59*** |
| **BEC** | 0.04 | -0.05 | 0.04 | -0.13 | -0.17* | -0.07 |  |

| **2010** | **SW** | **PH** | **SN** | **PN** | **BN** | **SY** | **BEC** |
| --- | --- | --- | --- | --- | --- | --- | --- |
| **SW** |  | 0.30*** | - | 0.33*** | - | 0.49*** | 0.34*** |
| **PH** | -0.19* |  | - | 0.34*** | - | 0.39*** | 0.29*** |
| **SN** | -0.41*** | 0.19* |  | - | - | - | - |
| **PN** | -0.22** | 0.53*** | 0.22** |  | - | 0.74*** | 0.65*** |
| **BN** | -0.36*** | 0.45*** | 0.18* | 0.47** |  | - | - |
| **SY** | -0.17* | 0.42*** | 0.69*** | 0.68*** | 0.30*** |  | 0.80*** |
| **BEC** | -0.02 | -0.25*** | 0.02 | -0.29*** | -0.32*** | -0.16* |  |

| **2011** | **SW** | **PH** | **SN** | **PN** | **BN** | **SY** | **BEC** |
| --- | --- | --- | --- | --- | --- | --- | --- |
| **SW** |  | 0.09 | 0.06 | 0.01 | -0.08 | 0.13 | 0.01 |
| **PH** | -0.44*** |  | 0.16* | 0.24*** | 0.26*** | 0.16* | -0.13 |
| **SN** | 0.28*** | -0.42*** |  | 0.32*** | -0.09 | 0.65*** | 0.44*** |
| **PN** | -0.51*** | 0.21** | -0.45*** |  | -0.02 | 0.52*** | 0.40*** |
| **BN** | 0.21** | -0.49*** | 0.33*** | -0.41*** |  | -0.06 | -0.10 |
| **SY** | -0.47*** | 0.13 | -0.54*** | 0.36*** | -0.45*** |  | 0.74*** |
| **BEC** | 0.90*** | -0.41*** | 0.21** | -0.51*** | 0.23** | -0.45*** |  |

Note:

The first table showed the Pearson’s correlation in the TNDH population. BEC, B efficiency coefficient; SYLB, seed yield under low B condition; SYNB, seed yield under normal B condition. “-1” and “-2” mean 2004 and 2005, respectively. * P<0.05, ** P<0.01, *** P<0.001.

The latter three tables showed the Pearson’s correlation in the BQDH population. Each one of them represented correlation of one trial. The data above diagonal of these three tables are the correlation coefficients under low B condition, and the data below diagonal are the correlation coefficients under normal B condition. SW, seed weight; PH, plant height; SN, seed number; PN, pot number per plant; BN, branch number; SY, seed yield; BEC, boron efficiency coefficient.
