# Supplementary material for: Dissecting Quantitative Trait Loci for Boron Efficiency across Multiple Environments in Brassica napus
Source: PLoS One. 2012 Sep 24;7(9):e45215. doi: 10.1371/journal.pone.0045215 (PMC3454432; doi:10.1371/journal.pone.0045215)
Supplement: Table S3 — Epistatic interactions for six yield related traits under both B conditions and BEC in BQDH population of Brasscia napus. (DOCX) [file pone.0045215.s005.docx]

**Table S3** Epistatic interactions for six yield related traits under both B conditions and BEC in BQDH population of *Brasscia napus*

| **Trait^a^** | **Type^b^** | **QTL_i** | **interval-i** | **pos_i** | **range_i** | **QTL_j** | **interval-j** | **pos_j** | **range_j** | **AA** | **AAE1** | **AAE2** | **PVE% (AA)** | **PVE% (AAE)** |
| --- | --- | --- | --- | --- | --- | --- | --- | --- | --- | --- | --- | --- | --- | --- |
| PNNB | QtQn | 7-11 | BRMS005-sR7223b | 67.5 | 59.5-67.7 | 16-4 | **BoGMS1497-O6Au-4** | 15.3 | 5.3-28.8 | 7.24*** |  |  | 1.62 |  |
| SYLB | QnQn | 1-12 | ea10-BoGMS0789 | 41.3 | 33.6-47.5 | 10-4 | BoGMS0949-sORH13a | 7.4 | 0.0-9.1 | 0.29*** | 0.34** |  | 3.34 | 3.13 |
|  | QnQn | 3-31 | BnGMS584b-CNU370 | 103.7 | 100.7-109.3 | 15-6 | BoGMS0319-S010I09-C4-1c | 54.2 | 54.2-60.2 | -0.19** | -0.29* |  | 0.75 | 1.17 |
|  | QnQn | 7-9 | BRAS023-BRMS018 | 45.2 | 41.7-47.2 | 7-21 | Nip5;1-Br3a-BoGMS0721c | 99.2 | 88.8-99.2 | -0.33*** | -0.36** | 0.33** | 2.77 | 3.3 |
|  | QnQn | 7-21 | Nip5;1-Br3a-BoGMS0721c | 99.2 | 88.8-99.2 | 16-5 | O6Au-4-BoGMS0347b | 23.2 | 14.3-26.5 | 0.29*** |  |  | 2.89 |  |
|  | QnQn | 9-21 | BoGMS0603-Na10G06 | 69 | 62.0-73.2 | 10-11 | H034P05-CB10524 | 18.2 | 14.3-20.2 | -0.27*** |  |  | 1.57 |  |
|  | QnQn | 12-13 | B060E11-1-sR94102b | 17.6 | 3.5-21.6 | 17-2 | sN0706-BRAS019 | 1.8 | 0.0-11.6 | 0.35*** | 0.22* |  | 3.42 | 2.06 |
| SYNB | QnQn | 2-14 | BeA2ssr09-BeA2ssr11 | 50.5 | 39.9-52.9 | 13-1 | CB10079b-CB10358 | 2 | 0.0-9.0 | -0.47*** |  |  | 2.22 |  |
|  | QnQn | 2-28 | B070J11-2-BRAS011a | 60 | 59.6-60.6 | 7-1 | HAU34-1-sR0282Ra | 7 | 0.0-13.0 | 0.88*** |  |  | 5.67 |  |
|  | QnQn | 6-5 | CNU325a-BnGMS331 | 51.7 | 44.7-58.5 | 12-3 | BoGMS1290-HBr024 | 6.2 | 3.5-9.2 | 0.84*** |  |  | 6.1 |  |
|  | QtQn | 7-16 | niab043-BoGMS1530 | 74.7 | 71.9-77.2 | 16-7 | **BRMS036-BoGMS0742** | 26.8 | 25.5-27.8 | 0.99*** |  |  | 14.26 |  |
| SNLB | QnQn | 7-17 | BoGMS1530-CNU167 | 75.1 | 71.3-77.9 | 16-7 | BRMS036-BoGMS0742 | 27.8 | 24.5-30.5 | 1.46*** |  |  | 8.94 |  |
| SNNB | QtQt | 2-41 | **OL12B03a-BeA2ssr38** | 83.9 | 80.0-84.9 | 7-20 | **CNU331-Nip5;1-Br3a** | 87.8 | 79.9-95.8 | -0.68*** |  |  | 1.15 |  |
|  | QnQn | 1-4 | niab071-BP2607-Br1 | 19.4 | 14.5-21.5 | 12-1 | sR12095a-B2-S2-1 | 0 | 0.0-2.0 | -1.12*** |  |  | 4.54 |  |
|  | QnQn | 2-20 | em5me28d-BoGMS0659 | 57.5 | 53.9-59.6 | 4-1 | CB10493a-BoGMS0252b | 0 | 0.0-3.0 | 1.00*** |  |  | 3.33 |  |
|  | QnQn | 4-1 | CB10493a-BoGMS0252b | 0 | 0.0-3.0 | 18-14 | CB10373-BoGMS1377 | 51 | 47.5-53.0 | -0.57*** |  |  | 0.53 |  |
|  | QnQn | 7-4 | BoGMS1575-BoGMS0531 | 19.4 | 15.6-24.4 | 12-6 | BoGMS1123-BeA2ssr02 | 12.9 | 10.5-17.3 | 0.89*** |  |  | 3.08 |  |
|  | QtQn | 7-15 | MK220-niab043 | 71.9 | 70.3-74.7 | 16-4 | **BoGMS1497-O6Au-4** | 15.3 | 11.3-20.3 | 1.01*** |  |  | 4.8 |  |
| BNNB | QtQt | 7-19 | **niab030-CNU331^c^** | 77.9 | 73.7-80.8 | 19-4 | **FITO516-HBr303b** | 31.4 | 25.1-32.2 |  | -0.22* |  |  | 1.74 |
| BEC | QnQn | 5-32 | H026E14b-CB10080 | 73.3 | 72.6-75.3 | 16-2 | Nip5;1-Br3b-CNU053b | 2 | 0.0-16.3 | 0.04*** | 0.04** |  | 3.26 | 2.55 |
|  | QnQn | 11-5 | BoGMS0292-BoGMS0794 | 20.2 | 16.0-23.7 | 18-17 | BoGMS1460-ew1 | 80.3 | 73.7-83.5 | 0.05*** | 0.05*** | -0.03* | 3.84 | 3.68 |
| SWLB | QtQn | 7-18 | **CNU167-niab030** | 76.2 | 75.1-80.8 | 16-7 | BRMS036-BoGMS0742 | 26.8 | 24.5-29.5 | 0.17*** |  |  | 7.16 |  |
| SWNB | QtQn | 7-6 | HAU49-4-BRMS040 | 32.7 | 21.4-41.7 | 10-11 | **H034P05-CB10524** | 26.2 | 22.2-29.9 | -0.06*** |  |  | 0.61 |  |
|  | QtQt | 16-4 | **BoGMS1497-O6Au-4** | 16.3 | 12.3-21.3 | 19-4 | **FITO516-HBr303b** | 30.4 | 27.1-32.2 | -0.07*** |  |  | 1.43 |  |
|  | QnQn | 6-14 | BRMS030-BnGMS132 | 74.6 | 64.8-77.6 | 16-2 | Nip5;1-Br3b-CNU053b | 2 | 0.0-5.1 | 0.08*** |  |  | 2.25 |  |
|  | QtQn | 7-2 | sR0282Ra-sR0282Rb | 15 | 7.0-16.6 | 16-7 | **BRMS036-BoGMS0742** | 26.8 | 25.5-28.8 | 0.10*** |  |  | 4.25 |  |
|  | QtQn | 13-33 | FITO314b-FITO094 | 193.8 | 187.8-193.8 | 16-4 | **BoGMS1497-O6Au-4** | 16.3 | 12.3-21.3 | 0.10*** |  |  | 3.48 |  |
| PHLB | QnQn | 14-3 | BRAS072b-BoGMS0252a | 8.1 | 4.7-14.8 | 15-9 | BnGMS433-BoGMS1297 | 69.3 | 66.0-76.5 | -3.97*** |  |  | 3.93 |  |
| PHNB | QnQn | 3-1 | B055N13-3-niab115a | 1 | 0.0-8.0 | 6-4 | CNU400-CNU325a | 26.8 | 18.6-29.8 | 2.33*** |  |  | 0.63 |  |
|  | QtQn | 3-1 | B055N13-3-niab115a | 1 | 0.0-8.0 | 13-6 | **BoGMS0953-BoGMS1288** | 34.2 | 26.3-38.3 | -4.53*** |  |  | 4.12 |  |
|  | QnQn | 4-8 | sN11516-BoGMS0798 | 43.4 | 42.3-45.8 | 5-10 | sN12353-BoGMS0033 | 47.1 | 46.0-47.8 | 3.16*** |  |  | 4.44 |  |
|  | QtQn | 7-20 | **CNU331-Nip5;1-Br3a** | 89.8 | 80.8-97.8 | 16-7 | BRMS036-BoGMS0742 | 26.8 | 24.5-28.8 | 3.62*** |  |  | 2.67 |  |

a: LB, low B condition; NB, normal B condition; PN, pot number per plant; SY, seed yield; SN, seed number; BN, branch number; BEC, B efficiency coefficient; SW, seed weight; PH, plant height.

b: epistatic interaction type based on the two locus involved, Qt meaned QTL with significant main effect, Qn meaned QTL.

c: regions with boldfaced letters meaned that the loci of epistatic interactions were located in main effect QTL intervals.

Significance: * *P*<0.05; ** *P*<0.01; *** *P*<0.001.

AA, additive×additive epstatic effect, AAE, additive×additive by environment interactive effect.

PVE, phenotypic variation explained.
