# Supplementary material for: Dissecting Quantitative Trait Loci for Boron Efficiency across Multiple Environments in Brassica napus
Source: PLoS One. 2012 Sep 24;7(9):e45215. doi: 10.1371/journal.pone.0045215 (PMC3454432; doi:10.1371/journal.pone.0045215)
Supplement: Table S4 — Orthologous genes associated with yield and yield-associated QTLs detected in BQDH population by in-silico mapping between A.thaliana and B.napus. (DOCX) [file pone.0045215.s006.docx]

**Table S4** Orthologous genes associated with yield and yield-associated QTLs detected in BQDH population by *in silico* mapping between *A. thaliana* and *B. napus*.

| **Trait** | ***QTL*** | **Alignment to BQDH map (cM)** | **Gene information in *Arabidopsis*** | | | | | |
| --- | --- | --- | --- | --- | --- | --- | --- | --- |
|  |  |  | **Locus** | **Gene name** | **Chr.** | **Start (bp)** | **End (bp)** | **Traits involved** |
| SY | *SYNB-A1* | 28.85 | AT4G32810 | *MAX4/CCD8* | 4 | **15828232** | **15831489** | branch number |
|  |  | 31.08 | AT4G32510.1 | BOR6 | 4 | **15685903** | **15688811** | HCO3-transpoter family |
|  |  | 31.69 | AT4G32410 | *CESA1* | 4 | **15646671** | **15640626** | plant height |
|  |  | 33.84 | AT4G32040 | *KNAT5* | 4 | **15494071** | **15496362** | branch number |
|  |  | 35.62 | AT4G31500 | *CYP83B1* | 4 | **15275316** | **15273477** | branch number |
|  |  | 37.74 | AT4G30610 | *BRS1* | 4 | **14944135** | **14948611** | plant height |
|  |  | 38.51 | AT4G30270 | *BRU1/TSK* | 4 | **14820598** | **14819198** | branch number/plant height |
|  |  | 45.87 | AT4G27260 | *WES1* | 4 | **13653615** | **13656210** | branch number/plant height |
|  |  | 47.77 | AT4G26420 | *GAMT1* | 4 | **13352577** | **13350805** | plant height |
| BN | *BNLB-A1* | 45.87 | AT4G27260 | *WES1* | 4 | **13653615** | **13656210** | branch number/plant height |
| PN | *PNLB-A2* | 79.30 | At5g28540 | BIP1 | 5 | **10540460** | **10543375** | expresses induced by B starvation |
| SN | *SNNB-A2* |  |  |  |  |  |  |  |
| SY | *SYLB-A2a* | 76.48 | AT5G25220 | *KNAT3* | 5 | **8736077** | **8738587** | branch number |
|  | *SYLB-A2b* | 76.66 | AT5G25430.1 | BOR7 | 5 | **8851251** | **8854259** | HCO3-transpoter family |
|  |  | 76.95 | AT5G25900 | *GA3/KO* | 5 | **9036021** | **9038409** | plant height |
| PH | *PHLB-A3a* | 21.52 | AT5G13790 | *AGL15* | 5 | **4450846** | **4449017** | plant height |
|  | *PHNB-A3a* | 21.82 | AT5G14400 | *CYP724A1* | 5 | **4643524** | **4646385** | plant height |
|  |  | 22.29 | AT5G15230 | *GASA4* | 5 | **4944903** | **4946219** | plant height/seed weight/seed yield |
|  |  | 23.57 | AT5G17490 | *RGL3* | 5 | **5766079** | **5764065** | plant height |
|  |  | 23.84 | At5g17920 | ATCIMS | 5 | **5935038** | **5939487** | response to low B stress |
|  |  | 24.15 | AT2G32440 | *KAO2/CYP88A4* | 2 | **13782656** | **13785079** | plant height |
|  |  | 24.16 | AT2G32460 | *ATM1* | 2 | **13791548** | **13789285** | plant height |
|  |  | 24.22 | AT2G32590.1 | CAP-H | 2 | **13829420** | **13833209** | expresses induced by excess B |
| PH | *PHLB-A3b* | 33.35 | AT5G56300 | *GAMT2* | 5 | **22816733** | **22818918** | plant height |
|  | *PHNB-A3b* | 34.53 | AT5G58220 | *TTL* | 5 | **23573109** | **23571612** | plant height |
|  |  | 34.54 | AT5G58270 | *STA1* | 5 | **23579368** | **23584416** | plant height |
|  |  | 35.53 | AT2G32440 | *KAO2/CYP88A4* | 2 | **13782656** | **13785079** | plant height |
|  |  | 35.54 | AT2G32460 | *ATM1* | 2 | **13791548** | **13789285** | plant height |
|  |  | 35.59 | AT2G32590.1 | CAP-H | 2 | **13829420** | **13833209** | expresses induced by excess B |
| SW | *SWLB-A4* | 20.75 | AT2G37260 | *TTG2* | 2 | **15652486** | **15654007** | seed weight |
|  |  | 22.04 | AT2G36880.1 | MAT3 | 2 | **15479366** | **15481412** | biosynthetic process genes induced by low B |
| BN | *BNNB-A6* | 61.78 | AT3G49690 | *RAX3/MYB84* | 3 | **18438720** | **18440186** | branch number |
|  |  | 63.79 | AT1G20925 | *AT1G20925* | 1 | **7290601** | **7292496** | branch number |
|  |  | 65.18 | AT1G23080 | *PIN7* | 1 | **8183559** | **8180566** | branch number |
| SN | *SNNB-A6* | 86.10 | AT4G25420 | *GA5/ATGA20ox1* | 4 | **12992468** | **12990894** | plant height/seed number |
| BEC | *BEC-A7a* | 0.00 | AT2G18390 | *TTN5/ARL2* | 2 | **7995247** | **7996607** | seed weight |
|  |  | 0.00 | AT2G19450 | *TAG1* | 2 | **8007886** | **8004474** | seed weight |
|  |  | 0.00 | AT2G20120 | *COV1* | 2 | **8696557** | **8694435** | branch number |
|  |  | 0.00 | AT2G20180 | [*PIL5/PIF1*](http://www.arabidopsis.org/servlets/TairObject?id=1000641429&type=gene) | 2 | **8713973** | **8711105** | plant height |
|  |  | 0.00 | AT2G20190 | *CLASP* | 2 | **8726099** | **8718680** | plant height |
| SY | *SYNB-A7* | 18.17 | AT1G23080 | *PIN7* | 1 | **8183559** | **8180566** | branch number |
| BEC | *SYLB-A7* | 18.36 | AT1G23380 | *KNAT6* | 1 | **8302640** | **8297280** | branch number |
|  | *BEC-A7b* | 19.37 | AT1G25490 | *RCN1* | 1 | **8951189** | **8955094** | branch number |
|  |  | 19.49 | AT2G17800 | *ROP3* | 2 | **7746938** | **7749238** | branch number |
|  |  | 19.87 | AT2G18390 | *TTN5/ARL2* | 2 | **7995247** | **7996607** | seed weight |
|  |  | 19.89 | AT2G19450 | *TAG1* | 2 | **8007886** | **8004474** | seed weight |
|  |  | 28.59 | AT1G23080 | *PIN7* | 1 | **8183559** | **8180566** | branch number |
|  |  | 28.77 | AT1G23380 | *KNAT6* | 1 | **8302640** | **8297280** | branch number |
|  |  | 29.78 | AT1G25490 | *RCN1* | 1 | **8951189** | **8955094** | branch number |
|  |  | 30.02 | AT1G26310 | *CAL* | 1 | **9103590** | **9100140** | branch number/flowering time |
|  |  | 30.18 | AT1G26630 | *FBR12* | 1 | **9205823** | **9207370** | plant height/seed number |
|  |  | 30.32 | AT1G26830 | *CUL3A* | 1 | **9295723** | **9298528** | seed weight |
|  |  | 30.62 | AT1G27320 | *AHK3* | 1 | **9487767** | **9492280** | plant height/seed weight |
|  |  | 31.14 | AT1G28130 | *F3H9.21* | 1 | **9825296** | **9828054** | branch number |
| SW | *SWLB-A7a* | 19.87 | AT2G18390 | *TTN5/ARL2* | 2 | **7995247** | **7996607** | seed weight |
|  |  | 19.89 | AT2G19450 | *TAG1* | 2 | **8007886** | **8004474** | seed weight |
|  |  | 30.32 | AT1G26830 | *CUL3A* | 1 | **9295723** | **9298528** | seed weight |
|  |  | 30.62 | AT1G27320 | *AHK3* | 1 | **9487767** | **9492280** | plant height/seed weight |
| PH | *PHLB-A7* | 73.28 | AT3G62270.1 | BOR2 | 3 | **23042284** | **23046048** | HCO3-transporter family |
|  |  | 73.70 | AT1G80350 | *LUE1* | 1 | **30213097** | **30210207** | plant height |
|  |  | 73.70 | AT1G80340 | *GA4H/GA3OX2* | 1 | **30207092** | **30205585** | plant height |
|  |  | 73.70 | AT1G80330 | *ATGA3ox4* | 1 | **30204429** | **30202953** | plant height |
| BN | *BNNB-A7* | 69.35 | AT3G54720 | *COP2/AMP1* | 3 | **20268826** | **20265703** | branch number/flowering time |
|  |  | 71.89 | AT3G59900 | *ARGOS* | 3 | **22140703** | **22141441** | branch number |
|  |  | 73.28 | AT3G62270.1 | BOR2 | 3 | **23042284** | **23046048** | HCO3-transporter family |
|  |  | 73.93 | AT1G79500.5 | AtkdsA1 | 1 | **29903420** | **29909437** | KDOP synthase related genes expressed under low B |
|  |  | 74.40 | AT1G77850 | *ARF17* | 1 | **29277207** | **29280313** | branch number |
|  |  | 74.63 | AT1G77110 | *PIN6* | 1 | **28975698** | **28979497** | branch number |
|  |  | 74.82 | AT1G76530 | *F14G6.13* | 1 | **28723273** | **28725265** | branch number |
|  |  | 74.82 | AT1G76520 | *F14G6.12* | 1 | **28719972** | **28722310** | branch number |
|  |  | 74.85 | AT1G76420 | *CUC3* | 1 | **28678828** | **28676923** | branch number |
|  |  |  |  |  |  |  |  |  |
| SW | *SWLB-A7b* | 73.28 | AT3G62270.1 | BOR2 | 3 | **23042284** | **23046048** | HCO3-transporter family |
|  | *SWNB-A7* | 73.93 | AT1G79500.5 | AtkdsA1 | 1 | **29903420** | **29909437** | KDOP synthase related genes expressed under low B |
|  |  | 75.28 | AT1G74810.1 | BOR5 | 1 | **28108474** | **28111534** | HCO3-transporter family |
|  |  | 82.40 | At1g70410 | ATBCA4 | 1 | **26533926** | **26536671** | response to low B stress |
|  |  | 82.56 | AT1G70070 | *ISE2/EMB25* | 1 | **26397859** | **26393679** | seed number/seed weight |
|  |  | 82.78 | AT1G69670 | *CUL3B* | 1 | **26207639** | **26205694** | seed weight |
| PH | *PHNB-A7* | 79.40 | AT1G73340 | T9L24.44 | 1 | **27576797** | **27578934** | plant height |
|  |  | 79.93 | AT1G73687 | MIR159A | 1 | **27717076** | **27716895** | plant height |
|  |  | 81.04 | AT1G73687 | MIR159A | 1 | **27717076** | **27716895** | plant height |
|  |  | 81.20 | AT1G73340 | T9L24.44 | 1 | **27576797** | **27578934** | plant height |
|  |  | 82.40 | At1g70410 | ATBCA4 | 1 | **26533926** | **26536671** | response to low B stress |
| SN | *SNNB-A7* | 82.40 | At1g70410 | ATBCA4 | 1 | **26533926** | **26536671** | response to low B stress |
|  |  | 82.56 | AT1G70070 | *ISE2/EMB25* | 1 | **26397859** | **26393679** | seed number/seed weight |
|  |  | 83.23 | AT1G68725 | *ATAGP19* | 1 | **25812961** | **25813793** | branch number/pod number/seed number |
|  |  | 98.15 | AT4G10380.1 | *NIP5;1* | 4 | **6431236** | **6434822** | boric acid channel protein |
| PN | *PNLB-A7c* | 98.15 | AT4G10380.1 | *NIP5;1* | 4 | **6431236** | **6434822** | boric acid channel protein |
| SW | *SWNB-A9* | 67.51 | AT1G31885.1 | NIP3;1 | 1 | **11450460** | **11451985** | water channel transport family |
|  |  | 68.33 | AT1G35720.1 | ANNEXIN | 1 | **13225197** | **13227195** | abiotic stress related genes induced by low B |
|  |  | 72.40 | AT4G18910.1 | NIP1;2 | 4 | **10366059** | **10368381** | water channel transport family |
| SW | *SWNB-A10* | 18.34 | AT5G59290.2 | ATUXS3 | 5 | **23915530** | **23917998** | response to low B stress |
|  |  | 35.36 | At5g17920 | ATCIMS | 5 | **5935038** | **5939487** | response to low B stress |
| PH | *PHNB-C3* | 26.06 | AT5G05170 | *CESA3* | 5 | **1535415** | **1530175** | plant height |
|  | *PHLB-C3b* | 26.19 | AT5G05690 | *DWF3/CPD* | 5 | **1706788** | **1702689** | plant height |
|  |  | 26.29 | AT5G06100 | *MYB33* | 5 | **1837908** | **1840728** | plant height |
|  |  | 31.14 | AT5G07200 | *YAP169/GA20OX3* | 5 | **2245340** | **2243554** | plant height |
|  |  | 31.62 | AT5G08130 | *BIM1* | 5 | **2609607** | **2606201** | plant height |
|  |  | 31.90 | AT5G08670.1 | AT5G08670 | 5 | **2818117** | **2821176** | expresses under low B condition |
|  |  | 33.16 | At5g11670 | NADP-ME2 | 5 | **3754249** | **3758242** | cell structure related genes induced by low B |
|  |  | 34.10 | AT5G13790 | *AGL15* | 5 | **4450846** | **4449017** | plant height |
|  |  | 34.36 | AT5G14400 | *CYP724A1* | 5 | **4643524** | **4646385** | plant height |
|  |  | 34.76 | AT5G15230 | *GASA4* | 5 | **4944903** | **4946219** | plant height/seed weight/seed yield |
|  |  | 35.86 | AT5G17490 | *RGL3* | 5 | **5766079** | **5764065** | plant height |
|  |  | 36.09 | At5g17920 | ATCIMS | 5 | **5935038** | **5939487** | response to low B stress |
|  |  | 36.25 | AT5G18280 | *APY2* | 5 | **6054343** | **6050516** | plant height |
|  |  | 36.64 | AT5G18930 | *BUD2* | 5 | **6313325** | **6311727** | branch number/plant height |
|  |  | 37.07 | AT5G19530 | *ACL5* | 5 | **6591365** | **6588960** | branch number/plant height |
| SY | *SYLB-C3* | 65.95 | AT2G32410 | *AXL* | 2 | **13764670** | **13768154** | branch number |
| BEC | *BEC-C3* | 66.03 | AT2G32440 | *KAO2/CYP88A4* | 2 | **13782656** | **13785079** | plant height |
|  |  | 66.07 | AT2G32460 | *ATM1* | 2 | **13791548** | **13789285** | plant height |
|  |  | 66.25 | AT2G32590.1 | CAP-H | 2 | **13829420** | **13833209** | expresses induced by excess B |
|  |  | 67.30 | AT2G33150 | *KAT2* | 2 | **14058217** | **14054555** | pod number/seed number |
|  |  | 68.58 | AT2G33860 | *ARF3/ETT* | 2 | **14336054** | **14332345** | branch number |
|  |  | 69.40 | AT2G34390.1 | NIP2;1 | 2 | **14514487** | **14515915** | water channel transport family |
|  |  | 69.63 | AT2G34555 | *ATGA2ox3* | 2 | **14564067** | **14565776** | plant height |
|  |  | 69.73 | AT2G34630 | *GPPS/GPS1* | 2 | **14585619** | **14588806** | plant height/seed number |
|  |  | 69.79 | AT2G34650 | *PID* | 2 | **14598867** | **14596851** | plant height/branch number |
|  |  | 70.04 | AT2G34710 | *PHB* | 2 | **14651352** | **14646402** | branch number |
|  |  | 70.91 | AT2G35230 | *IKU1* | 2 | **14842293** | **14844434** | seed weight |
|  |  | 71.64 | AT2G35670 | *FIS2* | 2 | **14999644** | **15003835** | seed weight |
|  |  | 73.63 | AT2G36800 | *DOGT1* | 2 | **15432095** | **15430459** | plant height |
|  |  | 73.73 | AT2G36830 | *GAMMA-TIP1* | 2 | **15452504** | **15453653** | plant height |
|  |  | 73.85 | AT2G36880.1 | MAT3 | 2 | **15479366** | **15481412** | biosynthetic process genes induced by low B |
|  |  | 73.91 | AT2G36890 | *RAX2* | 2 | **15492858** | **15494356** | branch number |
|  |  | 73.99 | AT2G36910 | *ATPGP1* | 2 | **15509093** | **15514399** | branch number |
|  |  | 74.65 | AT2G37260 | *TTG2* | 2 | **15652486** | **15654007** | seed weight |
|  |  | 75.86 | AT2G38050 | *DET2/DWF6* | 2 | **15929391** | **15928214** | plant height |
|  |  | 76.05 | AT2G38120 | *AUX1/MAP1* | 2 | **15980071** | **15984258** | branch number |
| BN | *BNNB-C3b* | 80.70 | AT2G39885 | *MIR393A* | 2 | **16652101** | **16652233** | branch number |
|  |  | 82.43 | AT2G42620 | *MAX2* | 2 | **17763217** | **17765545** | branch number |
|  |  | 83.68 | AT2G44990 | *MAX3/CCD7* | 2 | **18566013** | **18568408** | branch number/plant height |
|  |  | 83.79 | AT2G45190 | *YAB1/FIL* | 2 | **18637786** | **18635339** | branch number |
|  |  | 84.86 | AT2G47000 | *PGP4* | 2 | **19321819** | **19316937** | branch number |
|  |  | 84.91 | AT2G47160.2 | BOR1 | 2 | **19357478** | **19361147** | boron transporter |
|  |  | 85.09 | AT2G47430 | *CKI1* | 2 | **19471157** | **19466115** | branch number |
| PH | *PHLB-C4* | 40.29 | AT2G38050 | *DET2/DWF6* | 2 | **15929391** | **15928214** | plant height |
|  |  | 40.99 | AT2G36880.1 | MAT3 | 2 | **15479366** | **15481412** | biosynthetic process genes induced by low B |
|  |  | 41.04 | AT2G36830 | *GAMMA-TIP1* | 2 | **15452504** | **15453653** | plant height |
|  |  | 41.07 | AT2G36800 | *DOGT1* | 2 | **15432095** | **15430459** | plant height |
|  |  | 42.04 | AT1G62300.1 | WRKY6 | 1 | **23016683** | **23019279** | cellular response to B starvation |
|  |  | 42.11 | AT1G62360 | *STM* | 1 | **23065387** | **23062248** | branch number/flowering time/plant height |
|  |  | 42.59 | AT1G63030 | *DDF2* | 1 | **23372075** | **23371072** | plant height |
| PH | *PHNB-C4* | 60.30 | AT5G17490 | *RGL3* | 5 | **5766079** | **5764065** | plant height |
|  |  | 60.57 | At5g17920 | ATCIMS | 5 | **5935038** | **5939487** | response to low B stress |
|  |  | 60.75 | AT5G18280 | *APY2* | 5 | **6054343** | **6050516** | plant height |
|  |  | 61.16 | AT5G18930 | *BUD2* | 5 | **6313325** | **6311727** | branch number/plant height |
| PN | *PNLB-C5* | 51.18 | AT1G15460.1 | BOR4 | 1 | **5310001** | **5314094** | HCO3-transporter family |
|  |  | 52.67 | AT1G16340.1 | ATKDSA2 | 1 | **5587893** | **5590633** | KDOP synthase related genes expressed under low B |
| PH | *PHNB-C6* | 0.00 | AT4G08920 | *CRY1* | 4 | **5724119** | **5727250** | plant height |
|  |  | 0.00 | AT4G10380.1 | NIP5;1 | 4 | **6431236** | **6434822** | boron uptake |
|  |  | 2.33 | AT1G66350 | *RGL1* | 1 | **24751858** | **24753705** | plant height |
|  |  | 3.05 | At1g70410 | ATBCA4 | 1 | **26533926** | **26536671** | response to low B stress |
|  |  | 3.47 | AT1G73340 | *T9L24.44* | 1 | **27576797** | **27578934** | plant height |
|  |  | 3.53 | AT1G73687 | *MIR159A* | 1 | **27717076** | **27716895** | plant height |
|  |  | 3.67 | AT1G74670 | *F1M20.35* | 1 | **28056947** | **28057810** | plant height |
|  |  | 3.69 | AT1G74810.1 | BOR5 | 1 | **28108474** | **28111534** | HCO3-transporter family |
|  |  | 3.72 | AT1G75080 | *BZR1* | 1 | **28189165** | **28191736** | plant height |
|  |  | 3.93 | AT1G76490 | *HMG1* | 1 | **28700654** | **28703687** | plant height |
| PN | *PNNB-C6* | 10.54 | AT1G74810.1 | BOR5 | 1 | **28108474** | **28111534** | HCO3-transporter family |
|  |  | 18.72 | At1g70410 | ATBCA4 | 1 | **26533926** | **26536671** | response to low B stress |
|  |  | 22.47 | AT1G68725 | *ATAGP19* | 1 | **25812961** | **25813793** | branch number/pod number/seed number |
|  |  | 26.17 | At1g70410 | ATBCA4 | 1 | **26533926** | **26536671** | response to low B stress |
| SW | *SWNB-C6a* | 10.54 | AT1G74810.1 | BOR5 | 1 | **28108474** | **28111534** | HCO3-transporter family |
|  |  | 18.72 | At1g70410 | ATBCA4 | 1 | **26533926** | **26536671** | response to low B stress |
|  |  | 19.43 | AT1G70070 | *ISE2/EMB25* | 1 | **26397859** | **26393679** | seed number/seed weight |
|  |  | 20.42 | AT1G69670 | *CUL3B* | 1 | **26207639** | **26205694** | seed weight |
| SN | *SNLB-C6* | 10.54 | AT1G74810.1 | BOR5 | 1 | **28108474** | **28111534** | HCO3-transporter family |
|  | *SNNB-C6a* | 18.72 | At1g70410 | ATBCA4 | 1 | **26533926** | **26536671** | response to low B stress |
|  |  | 19.43 | AT1G70070 | *ISE2/EMB25* | 1 | **26397859** | **26393679** | seed number/seed weight |
|  |  | 22.47 | AT1G68725 | *ATAGP19* | 1 | **25812961** | **25813793** | branch number/pod number/seed number |
| BN | *BNNB-C6* | 6.03 | AT1G77110 | *PIN6* | 1 | **28975698** | **28979497** | branch number |
| SY | *SYNB-C6a* | 7.34 | AT1G76530 | *F14G6.13* | 1 | **28723273** | **28725265** | branch number |
|  |  | 7.36 | AT1G76520 | *F14G6.12* | 1 | **28719972** | **28722310** | branch number |
|  |  | 7.46 | AT1G76490 | *HMG1* | 1 | **28700654** | **28703687** | plant height |
|  |  | 7.57 | AT1G76420 | *CUC3* | 1 | **28678828** | **28676923** | branch number |
|  |  | 10.12 | AT1G75080 | *BZR1* | 1 | **28189165** | **28191736** | plant height |
|  |  | 10.54 | AT1G74810.1 | BOR5 | 1 | **28108474** | **28111534** | HCO3-transporter family |
|  |  | 10.80 | AT1G74670 | *F1M20.35* | 1 | **28056947** | **28057810** | plant height |
|  |  | 12.57 | AT1G73687 | *MIR159A* | 1 | **27717076** | **27716895** | plant height |
|  |  | 12.85 | AT1G73590 | *PIN1* | 1 | **27663334** | **27666839** | branch number |
|  |  | 13.30 | AT1G73340 | *T9L24.44* | 1 | **27576797** | **27578934** | plant height |
|  |  | 17.26 | AT1G71090 | *F23N20.8* | 1 | **26815874** | **26817792** | branch number |
|  |  | 17.62 | AT1G70940 | *PIN3* | 1 | **26746716** | **26750057** | branch number |
|  |  | 18.48 | AT1G70510 | *KNAT2* | 1 | **26580149** | **26586094** | branch number |
|  |  | 18.72 | At1g70410 | ATBCA4 | 1 | **26533926** | **26536671** | response to low B stress |
|  |  | 19.43 | AT1G70070 | *ISE2/EMB25* | 1 | **26397859** | **26393679** | seed number/seed weight |
|  |  | 19.65 | AT1G69960 | *PP2A* | 1 | **26354360** | **26352384** | branch number |
|  |  | 20.42 | AT1G69670 | *CUL3B* | 1 | **26207639** | **26205694** | seed weight |
|  |  | 21.55 | AT1G69120 | *AP1/AGL7* | 1 | **25989976** | **25985993** | branch number/flowering time |
|  |  | 22.27 | AT1G68800 | *BRC2/TCP12* | 1 | **25852068** | **25851115** | branch number |
|  |  | 22.47 | AT1G68725 | *ATAGP19* | 1 | **25812961** | **25813793** | branch number/pod number/seed number |
|  |  | 26.17 | At1g70410 | ATBCA4 | 1 | **26533926** | **26536671** | response to low B stress |
|  |  | 28.63 | AT1G74810.1 | BOR5 | 1 | **28108474** | **28111534** | HCO3-transporter family |
| SW | *SWLB-C6* | 28.63 | AT1G74810.1 | BOR5 | 1 | **28108474** | **28111534** | HCO3-transporter family |
|  | *SWNB-C6b* | 31.43 | AT1G79500.5 | AtkdsA1 | 1 | **29903420** | **29909437** | KDOP synthase related genes expressed under low B |
|  |  | 37.71 | AT4G10380.1 | NIP5;1 | 4 | **6431236** | **6434822** | boric acid channel protein |
| SN | *SNNB-C6b* | 31.43 | AT1G79500.5 | AtkdsA1 | 1 | **29903420** | **29909437** | KDOP synthase related genes expressed under low B |
| SY | *SYNB-C6b* | 25.05 | AT1G68725 | *ATAGP19* | 1 | **25812961** | **25813793** | branch number/pod number/seed number |
|  |  | 25.11 | AT1G68800 | *BRC2/TCP12* | 1 | **25852068** | **25851115** | branch number |
|  |  | 25.32 | AT1G69120 | *AP1/AGL7* | 1 | **25989976** | **25985993** | branch number/flowering time |
|  |  | 25.66 | AT1G69670 | *CUL3B* | 1 | **26207639** | **26205694** | seed weight |
|  |  | 25.89 | AT1G69960 | *PP2A* | 1 | **26354360** | **26352384** | branch number |
|  |  | 25.96 | AT1G70070 | *ISE2/EMB25* | 1 | **26397859** | **26393679** | seed number/seed weight |
|  |  | 26.17 | At1g70410 | ATBCA4 | 1 | **26533926** | **26536671** | response to low B stress |
|  |  | 26.24 | AT1G70510 | *KNAT2* | 1 | **26580149** | **26586094** | branch number |
|  |  | 26.50 | AT1G70940 | *PIN3* | 1 | **26746716** | **26750057** | branch number |
|  |  | 26.61 | AT1G71090 | *F23N20.8* | 1 | **26815874** | **26817792** | branch number |
|  |  | 27.80 | AT1G73340 | *T9L24.44* | 1 | **27576797** | **27578934** | plant height |
|  |  | 27.93 | AT1G73590 | *PIN1* | 1 | **27663334** | **27666839** | branch number |
|  |  | 28.02 | AT1G73687 | *MIR159A* | 1 | **27717076** | **27716895** | plant height |
|  |  | 28.55 | AT1G74670 | *F1M20.35* | 1 | **28056947** | **28057810** | plant height |
|  |  | 28.63 | AT1G74810.1 | BOR5 | 1 | **28108474** | **28111534** | HCO3-transporter family |
|  |  | 28.75 | AT1G75080 | *BZR1* | 1 | **28189165** | **28191736** | plant height |
|  |  | 29.52 | AT1G76420 | *CUC3* | 1 | **28678828** | **28676923** | branch number |
|  |  | 29.55 | AT1G76490 | *HMG1* | 1 | **28700654** | **28703687** | plant height |
|  |  | 29.58 | AT1G76520 | *F14G6.12* | 1 | **28719972** | **28722310** | branch number |
|  |  | 29.59 | AT1G76530 | *F14G6.13* | 1 | **28723273** | **28725265** | branch number |
|  |  | 29.98 | AT1G77110 | *PIN6* | 1 | **28975698** | **28979497** | branch number |
|  |  | 30.45 | AT1G77850 | *ARF17* | 1 | **29277207** | **29280313** | branch number |
|  |  |  |  |  |  |  |  |  |
| SY | *SYLB-C7* | 40.81 | AT2G01420 | *PIN4* | 2 | **183327** | **180104** | branch number |
|  |  | 40.93 | AT2G01570 | *RGA/RGA1* | 2 | **257549** | **255248** | branch number/flowering time/plant height |
|  |  | 41.10 | AT2G01830 | *CRE1/AHK4* | 2 | **369706** | **362982** | branch number/plant height/seed weight |
|  |  | 47.44 | AT3G26790 | *FUS3* | 3 | **9857226** | **9855065** | plant height |
|  |  | 48.23 | AT5G13790 | *AGL15* | 5 | **4450846** | **4449017** | plant height |
| SW | *SWLB-C8* | 15.44 | AT1G04690.1 | KAB1 | 1 | **1313564** | **1315750** | transport related genes induced by low B stress |
| BEC | *BEC-C8* | 15.74 | AT1G05160 | *CYP88A3/KAO1* | 1 | **1490946** | **1487377** | plant height |
|  |  | 15.76 | AT1G05180 | *AXR1* | 1 | **1501823** | **1498113** | branch number |
|  |  | 16.06 | AT1G05630 | *5PTASE13* | 1 | **1682317** | **1687362** | branch number |
|  |  | 16.21 | AT1G05850 | *POM1* | 1 | **1768694** | **1766502** | branch number/plant height |
|  |  | 17.75 | AT1G08465 | *YAB2* | 1 | **2675810** | **2679784** | branch number |
|  |  | 18.44 | AT1G09530 | *PIF3* | 1 | **3076584** | **3079541** | plant height |
|  |  | 18.45 | AT1G09540 | *MYB61* | 1 | **3086161** | **3087914** | seed weight |
|  |  | 18.48 | AT1G09570 | *PHYA* | 1 | **3100359** | **3095258** | plant height |
|  |  | 18.76 | AT1G10010 | *AAP8* | 1 | **3265978** | **3268728** | seed number/seed weight |
|  |  | 18.90 | AT1G10210 | *ATMPK1* | 1 | **3349221** | **3351182** | branch number |
|  |  | 20.50 | AT1G12610 | *DDF1* | 1 | **4291015** | **4289942** | plant height |
|  |  | 21.23 | AT1G14920 | *GAI/SLR1* | 1 | **5149221** | **5151349** | plant height |
|  |  | 21.30 | AT1G15360 | *WIN1* | 1 | **5283533** | **5284668** | branch number |
|  |  | 21.31 | AT1G15460.1 | BOR4 | 1 | **5310001** | **5314094** | boron transport |
|  |  | 21.33 | AT1G15550 | *GA4/ATGA3ox1* | 1 | **5346161** | **5344473** | plant height |
|  |  | 21.35 | AT1G15690 | *AVP1* | 1 | **5398985** | **5402728** | branch number |
|  |  | 21.44 | AT1G16340.1 | ATKDSA2 | 1 | **5587893** | **5590633** | KDOP synthase related genes expressed under low B |
|  |  | 21.45 | AT1G16400 | *CYP79F2* | 1 | **5605153** | **5607467** | branch number |
|  |  | 21.45 | AT1G16410 | *BUS1/CYP79F1* | 1 | **5608801** | **5611420** | branch number |
|  |  | 21.48 | AT1G16540 | *ABA3* | 1 | **5659408** | **5665442** | plant height |
|  |  | 21.56 | AT1G17060 | *CYP72C1/SOB7* | 1 | **5835449** | **5832090** | plant height/seed weight |
|  |  | 21.75 | AT1G18075 | *MIR159B* | 1 | **6220641** | **6220826** | plant height |
|  |  | 21.79 | AT1G18350 | *ATMKK7/BUD1* | 1 | **6315644** | **6316731** | branch number/plant height |
|  |  | 23.16 | AT1G50430 | *DWF5/LE* | 1 | **18689318** | **18685600** | plant height |
| SW | *SWNB-C9* | 28.95 | AT5G25430.1 | BOR7 | 5 | **8851251** | **8854259** | HCO3-transporter family |
|  |  | 31.10 | At5g28540 | BIP1 | 5 | **10540460** | **10543375** | expresses induced by B starvation |
|  |  | 32.06 | AT5G65800 | *ETO2* | 5 | **26350038** | **26347972** | seed weight |
| PH | *PHNB-C9a* | 28.95 | AT5G25430.1 | BOR7 | 5 | **8851251** | **8854259** | HCO3-transporter family |
|  |  | 29.24 | AT5G25900 | *GA3/KO* | 5 | **9036021** | **9038409** | plant height |
|  |  | 30.17 | AT5G27320 | *ATGID1c* | 5 | **9629095** | **9631213** | plant height |
|  |  | 31.10 | At5g28540 | BIP1 | 5 | **10540460** | **10543375** | expresses induced by B starvation |
|  |  | 31.50 | AT5G64330 | *NPH3/RPT3* | 5 | **25744563** | **25747584** | plant height |
|  |  | 31.51 | AT5G64340 | *SAC51* | 5 | **25750539** | **25747561** | plant height |
|  |  | 31.67 | AT5G64800 | *CLE21* | 5 | **25923309** | **25923629** | plant height |
|  |  | 32.22 | AT5G66350 | *SHI* | 5 | **26523795** | **26521693** | branch number/plant height |
| PN | *PNNB-C9* | 28.95 | AT5G25430.1 | BOR7 | 5 | **8851251** | **8854259** | HCO3-transporter family |
|  |  | 31.10 | At5g28540 | BIP1 | 5 | **10540460** | **10543375** | expresses induced by B starvation |
| BN | *BNNB-C9a* | 31.10 | At5g28540 | BIP1 | 5 | **10540460** | **10543375** | expresses induced by B starvation |
|  |  | 31.99 | AT5G65670 | *IAA9* | 5 | **26270884** | **26273694** | branch number |
|  |  | 32.12 | AT5G65980 | *K2A18.4* | 5 | **26409670** | **26411458** | branch number |
|  |  | 32.22 | AT5G66350 | *SHI* | 5 | **26523795** | **26521693** | branch number/plant height |
| BN | *BNNB-C9b* | 39.36 | AT1G59500 | *GH3.4* | 1 | **21860279** | **21858158** | branch number |
| BN | *BNLB-C9* | 52.73 | AT2G01420 | *PIN4* | 2 | **183327** | **180104** | branch number |
|  |  | 52.85 | AT2G01570 | *RGA/RGA1* | 2 | **257549** | **255248** | branch number/flowering time/plant height |
|  |  | 53.02 | AT2G01830 | *CRE1/AHK4* | 2 | **369706** | **362982** | branch number/plant height/seed weight |

Note:

SY, seed yield; BN, branch number; PN, pot number per plant; SN, seed number per pot; SW, seed weight; PH, plant height; BEC, boron efficiency coefficient.

QTL name comprised with B treatment, trait name and linkage group name, a b c … were used to distinguish QTLs for the same trait and B condition on the same linkage group, such as NBBN-C9a.
